# Hepatic TNFRSF12A promotes bile acid-induced hepatocyte pyroptosis through NFκB/Caspase-1/GSDMD signaling in cholestasis

**DOI:** 10.1038/s41420-023-01326-z

**Published:** 2023-01-23

**Authors:** Min Liao, Junwei Liao, Jiaquan Qu, Pan Shi, Ying Cheng, Qiong Pan, Nan Zhao, Xiaoxun Zhang, Liangjun Zhang, Ya Tan, Qiao Li, Jin-Fei Zhu, Jianwei Li, Chengcheng Zhang, Shi-Ying Cai, Jin Chai

**Affiliations:** 1 Department of Gastroenterology, The First Affiliated Hospital (Southwest Hospital), Third Military Medical University (Army Medical University), Chongqing, 400038 China; 2 Institute of Digestive Diseases of PLA, The First Affiliated Hospital (Southwest Hospital), Third Military Medical University (Army Medical University), Chongqing, 400038 China; 3 Cholestatic Liver Diseases Center and Center for Metabolic-Associated Fatty Liver Diseases, The First Affiliated Hospital (Southwest Hospital), Third Military Medical University (Army Medical University), Chongqing, 400038 China; 4 Central South University School of Sciences, Changsha, Hunan 410083 China; 5 Department of Medical Imaging Technology, Medical College of Jishou University, Jishou, Hunan 416000 China; 6 Queen Mary School, Nanchang University, Nanchang, Jiangxi 330031 China; 7 Institute of Hepatobiliary Surgery, Southwest Hospital, Third Military Medical University, Chongqing, 400038 China; 8 Department of Internal Medicine and Liver Center, Yale University School of Medicine, New Haven, CT 06520 USA

**Keywords:** Primary biliary cirrhosis, Apoptosis

## Abstract

Tumor necrosis factor receptor superfamily member-12A (TNFRSF12A) plays a critical role in inflammation and cell death. It is expressed in multiple tissues yet extremely low in normal liver. To date, little is known about its role in cholestasis. Therefore, we sought to delineate the role of TNFRSF12A in cholestasis and its underlying mechanisms. Human liver tissues were collected from patients with obstructive cholestasis (OC) or primary biliary cholangitis (PBC). *Tnfrsf12a* knockout (KO) mice were generated. Cholestasis was induced by bile-duct ligation (BDL) or 0.1% 5-diethoxycarbonyl-1,4-dihydrocollidine (DDC)-feeding. Human hepatoma PLC/PRF/5-*ASBT* and THP1 cell lines or primary mouse hepatocytes were used for mechanistic studies. Hepatic TNFRSF12A expression was markedly increased in OC or PBC patients. Genetic ablation of *Tnfrsf12a* in BDL- and 0.1%DDC-induced cholestatic mice significantly attenuated cholestatic liver injury with remarkable reduction of hepatocyte pyroptosis but without changing scores of necroptosis and apoptosis. Morphological features of hepatocyte pyroptosis and increased levels of pyroptosis-related proteins, NLRP3, cleaved-Caspase-1, and cleaved-GSDMD in OC patients and BDL-mice confirmed this observation. Further mechanistic studies revealed that bile acids (BAs) induced TNFRSF12A expression by enhancing the transcription factor c-JUN binding to the *TNFRSF12A* promoter and subsequently initiated hepatocyte pyroptosis by the NFκB/Caspase-1/GSDMD signaling. Interestingly, TWEAK, a typical ligand of TNFRSF12A, secreted by infiltrated macrophages in cholestatic livers, enhanced TNFRSF12A-induced hepatocyte pyroptosis. Taken together, we report, for the first time, that hepatic TNFRSF12A is dramatically increased in human cholestasis. Deletion of *TNFRSF12A* inhibits BAs-induced hepatocyte pyroptosis through the NFκB/Caspase-1/GSDMD signaling and thereby ameliorates cholestatic liver injury. As such, targeting TNFRSF12A could be a promising approach to treating cholestasis.

## Introduction

Cholestasis is characterized by excessive accumulation of intrahepatic bile acids (BAs), which results in liver injury and can progress to liver cirrhosis, and eventually liver failure [1–5]. However, the pathologic role of BA in liver injury remains hotly debated.

Pyroptosis was initially recognized as monocyte death mediated by Caspase-1 in response to bacterial insults and recently has been redefined as a new form of cell death featured by gasdermin (GSDMD)-mediated programmed necrosis without the cell type-specificity [6]. At present, two distinct molecular pathways have been linked to the occurrence of pyroptosis: a canonical pathway involving the activation of caspase-1 by the inflammasome and a non-canonical pathway involving the activation of caspase-4/5/11 by lipopolysaccharide (LPS), both of which can cleave GSDMD and release the cleaved Gasdermin-N domain (GSDMD-N) to punch holes in the cell membrane, and subsequently pyroptotic morphological changes of cell lysis including cell swelling and large bubbles blowing from the plasma membrane [6–8]. Recent studies have reported that the inflammasome is involved in chronic liver diseases, including alcoholic hepatitis, nonalcoholic steatohepatitis (NASH), and cholestatic liver injury [8–10]. A very recent study described a significant pyroptosis in murine and human cholestasis [e.g., primary biliary cholangitis (PBC)] [5]. Han D et al. recently also demonstrated that Sestrin2 attenuates cholestatic liver injury by reducing the reticulum stress and NLRP3 inflammasome-induced hepatocyte pyroptosis [11], suggesting a crucial role of hepatocyte pyroptosis in cholestasis. However, whether and how BAs induce hepatocyte pyroptosis to cause cholestatic liver injury has not yet been explored.

Tumor necrosis factor receptor superfamily member-12A (TNFRSF12A/fibroblast growth factor-inducible 14, FN14) is the specific binding receptor of tumor necrosis factor-like weak inducer of apoptosis (TWEAK), a secretory cytokine that shares a 93% homology of amino acids in humans and mice [12]. TNFRSF12A is ubiquitously and constitutively expressed in human tissues, such as the heart, placenta, lung, skeletal muscle, kidney, and pancreas [13–15]. It exerts an important role in cellular processes, including inflammatory responses, angiogenesis, cell proliferation, and cell death [16–19]. Interestingly, TNFRSF12A is nearly undetectable in normal adult livers but substantially increased in hepatic progenitor cells during chronic liver diseases, including NASH, alcoholic liver disease, and chronic hepatitis C [20, 21]. Recent studies also demonstrated that the expression of Tnfrsf12a is markedly elevated in hepatic progenitor cells in mouse models of cholestasis induced by BDL operation and 5-diethoxycarbonyl-1,4- dihydrocollidine (DDC) diet [22, 23], implying its distinct role in cholestatic liver injury. To date, however, the functional significance and regulatory mechanism of TNFRSF12A in hepatocytes and human cholestasis are poorly characterized.

In this study, we aimed to examine the expression of hepatic TNFRSF12A and delineate its functional role and regulatory mechanism in human cholestasis. We found that hepatic TNFRSF12A expression was dramatically increased in patients with PBC and obstructive cholestasis (OC) and positively associated with cholestatic liver injury. Genetic ablation of *Tnfrsf12a* in BDL- and 0.1%DDC-induced cholestatic mice attenuated cholestatic liver injury with a remarkable reduction of hepatocyte pyroptosis. Further mechanism studies revealed that BAs stimulated TNFRSF12A expression at the transcriptional level in hepatocytes and subsequently initiated hepatocyte pyroptosis via the NFκB/Caspase-1/GSDMD signaling pathway, which was furhter enhanced by its ligand TWEAK. These findings through conducting this study may advance our understanding of the molecular mechanism of cholestatic liver injury and thus may offer novel therapeutic targets to facilitate the development of treatment for cholestasis.

## Results

### Hepatic TNFRSF12A is remarkably elevated in cholestasis, and its high expression is positively correlated with serum levels of ALT and AST

**Fig. 1:**
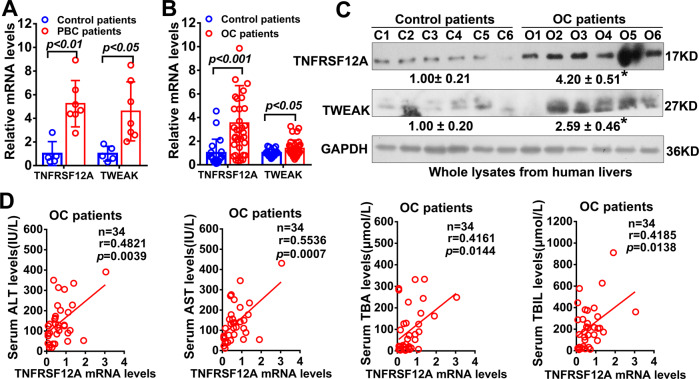
Hepatic TNFRSF12A expression and its correlation with cholestatic liver injury in cholestatic patients. **A** The levels of hepatic TNFRSF12A and TWEAK mRNA transcripts in control patients (*n* = 5) and PBC patients (*n* = 7); (**B**) The levels of hepatic TNFRSF12A and TWEAK mRNA transcripts in control patients (*n* = 20) and OC patients (*n* = 34); (**C**) Western blot analysis of TNFRSF12A and TWEAK protein levels in human liver tissues (C1-C6: control patients; O1-O6: OC patients). *p < *0.05* vs. control group; (**D**) Correlation analysis of TNFRSF12A mRNA and liver function tests in patients with OC. *p < *0.05* vs. control group.

Intrigued by the findings in BDL and 0.1%DDC-fed mice [22, 23], we examined the expression of TNFRSF12A in human cholestasis. As shown in Fig. 1A, the real-time qPCR analysis revealed that the levels of hepatic TNFRSF12A and its ligand TWEAK mRNA transcripts were remarkably increased in PBC patients compared to control patients. Similarly, we also detected significant hepatic TNFRSF12A and TWEAK expression increases at mRNA and protein levels in patients with OC (Fig. 1B, C).

We performed a correlation analysis between the TNFRSF12A mRNA levels and liver function tests to explore its functional role in human cholestasis. As shown in Fig.1D, there was a significantly positive correlation between the levels of hepatic TNFRSF12A mRNA transcripts and serum alanine aminotransferase (ALT), aspartate aminotransferase (AST), alkaline phosphatase (ALP), total bile acids (TBA), or total bilirubin (TBIL) in patients with OC. However, there was no correlation between hepatic TNFRSF12A mRNA levels and serum gamma-glutamyl transferase (GGT) or ALP levels in patients with cholestasis (Suppl. Fig. 2).

Furthermore, similar findings were observed in mouse models of cholestasis induced by BDL, 0.1%DDC feeding, or *Abcb4* gene deficiency, confirming the observations in patients with cholestasis. We found that hepatic levels of Tnfrsf12a and Tweak mRNA transcripts were also significantly increased in these cholestatic mouse models (Suppl. Fig. 1A–C).

Altogether, these findings indicated that hepatic TNFRSF12A expression was remarkably induced in cholestasis, and its dramatic increase may contribute to cholestatic liver injury.

### Genetic deletion of Tnfrsf12a attenuates cholestatic liver injury without enhancing hepatocyte necroptosis or apoptosis

**Fig. 2:**
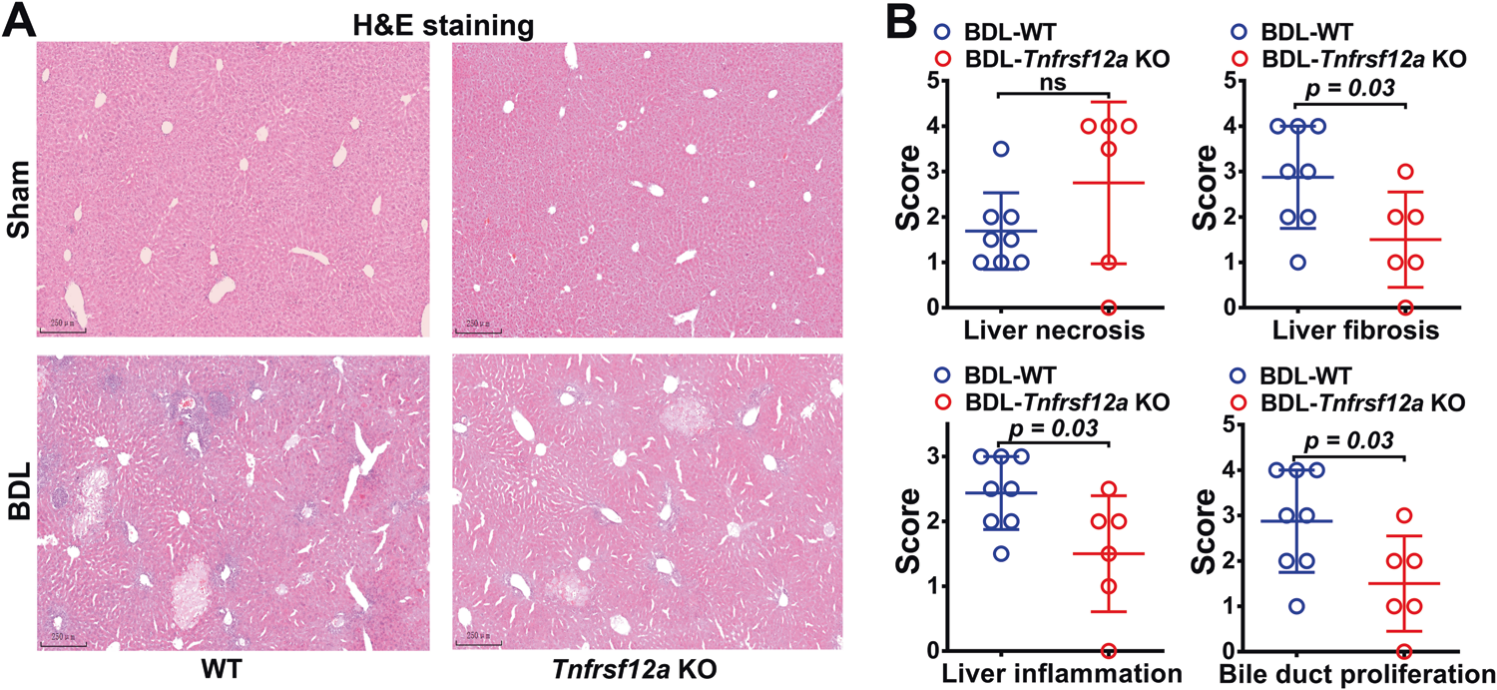
Effects of *Tnfrsf12a* deletion on BDL-induced obstructive cholestasis in mice. **A** Representative H&E staining images; **B** Liver histological scores of liver necrosis, fibrosis, inflammation, and bile duct proliferation in BDL-*Tnfrsf12a*-KO and BDL-WT mice. Liver histological assessments were performed by expert pathologists who were blinded to the group assignment.

To investigate the functional role of TNFRSF12A in cholestatic liver injury, *Tnfrsf12a* KO mice were generated (Suppl. Fig. 3), and cholestasis was induced by BDL or 0.1%DDC diet. After seven days of BDL, serum liver biochemistry analysis revealed that serum ALT, AST, TBIL, and TBA levels were significantly lower in *Tnfrsf12a* KO mice versus WT controls (Table 1). Similar results were observed in mice with 0.1%DDC feeding (Table S5). These data suggested that *Tnfrsf12a* deficiency alleviated cholestatic liver injury. However, liver histology stained with H&E showed no significant difference in necrotic infarction between BDL-*Tnfrsf12a* KO and BDL-WT mice, although histologic scores of liver fibrosis, inflammation, and bile-duct proliferation were significantly decreased in BDL-*Tnfrsf12a* KO compared to BDL-WT mice (Fig. 2A, B). Given other cell deaths in cholestasis, we next measured the apoptotic morphology in these mice. TUNEL-positive nuclei undergoing cell apoptosis in situ also demonstrated no significant difference in apoptotic cell rates between *Tnfrsf12a* KO and WT mice after BDL (Suppl. Fig. 4). These data supported that hepatic necroptosis and apoptosis were not significant contributors of *TNFRSF12A* deficiency-attenuated cholestatic liver injury, and that other cell death mechanisms might be involved.

**Table 1:** Serum biochemistry in WT and *Tnfrsf12a* KO mice with BDL for 7 days.

	Sham operation, 7 days		BDL operation, 7 days	
Liver function tests	WT (*n* = 8)	*Tnfrsf12a* KO (*n* = 8)	WT (*n* = 8)	*Tnfrsf12a* KO (*n* = 6)
Serum ALT (IU/L)	30.85 ± 10.23	25.10 ± 9.54	270.15 ± 127.46^*¶^	120.07 ± 58.72^*¶§^
Serum AST (IU/L)	88.50 ± 38.72	91.00 ± 33.78	596.95 ± 368.34^*¶^	221.13 ± 69.60^*¶§^
Serum ALP (IU/L)	129.50 ± 62.25	148.00 ± 75.50	540.50 ± 173.82^*¶^	351.67 ± 87.60^*¶§^
Serum TBA (μmol/L)	33.30 ± 47.33	9.90 ± 12.86	226.23 ± 117.47^*¶^	174.79 ± 203.35^¶^
Serum TBIL (μmol/L)	3.30 ± 2.29	3.78 ± 3.54	125.65 ± 79.23^*¶^	62.20 ± 55.46^*¶^
Serum DBIL (μmol/L)	1.34 ± 2.72	1.87 ± 3.46	88.83 ± 58.52^*¶^	30.49 ± 30.24^*¶§^

*BDL* bile duct ligation, *KO* Knock out, *ALT* alanine aminotransferase, *AST* aspartate aminotransferase, *ALP* alkaline phosphatase, *TBA* total bile salts, *TBIL* total bilirubin, *DBIL* Direct bilirubin.

Values are means ± SD. *P < 0.05 VS Sham-WT mice; ^¶^*P* < 0.05 VS Sham-*Tnfrsf12a* KO mice; ^§^*P* < 0.05 VS BDL-WT mice.

### Genetic ablation of Tnfrsf12a ameliorates cholestasis-induced hepatocyte pyroptosis in mice

**Fig. 3:**
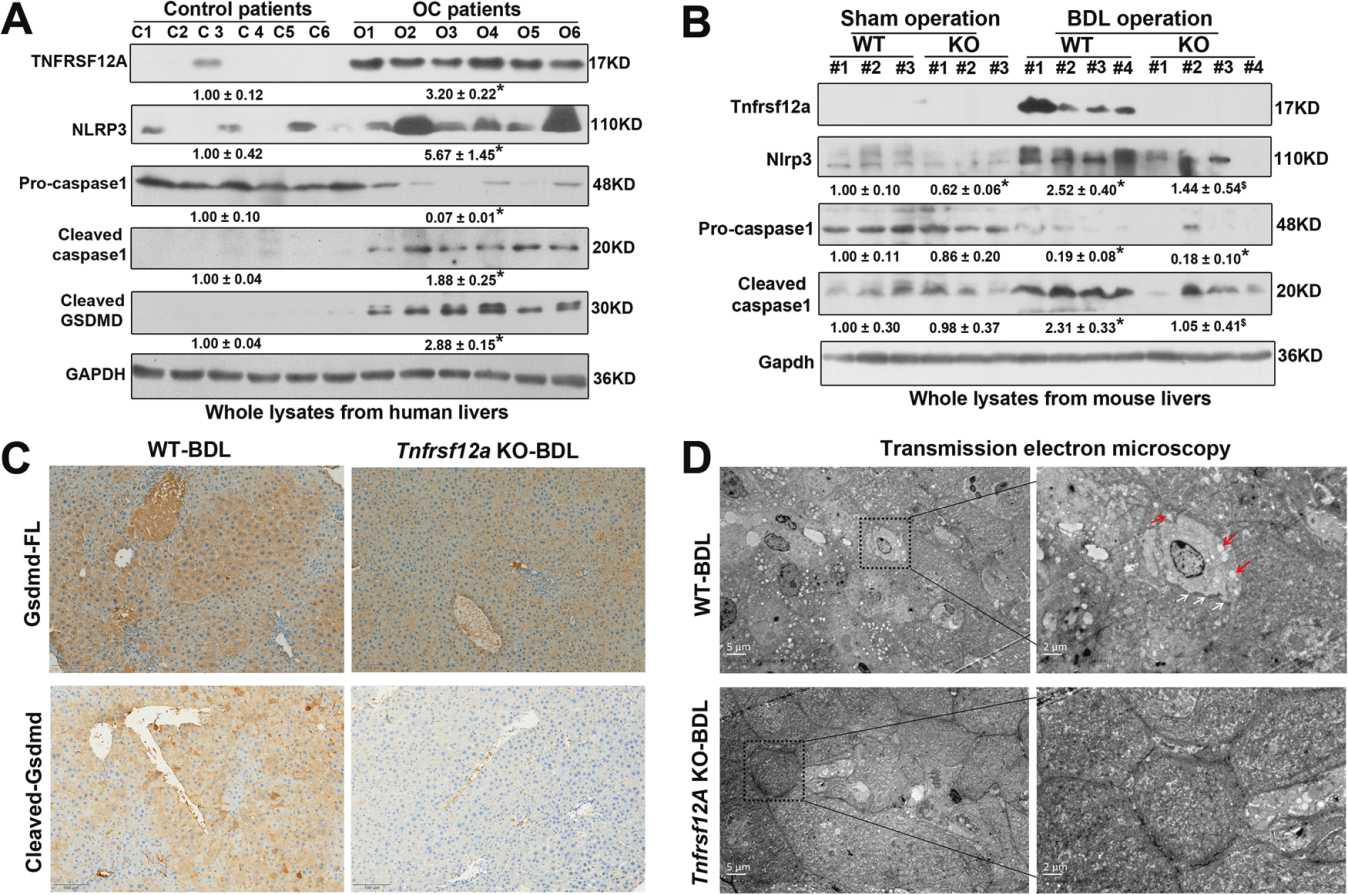
Effects of *Tnfrsf12a* on hepatocyte pyroptosis in human and experimental cholestasis. **A** Western blot analysis for TNFRSF12A, NLRP3, pro-casepase1, cleaved-Caspase-1, and cleaved-GSDMD in human control and obstructive cholestatic livers. *p < *0.05* vs. control group; **B** Western blot analysis of Tnfrsf12a, Nlrp3, pro-Caspase-1, and cleaved-Caspase-1 in WT and *Tnfrsf12a* KO mice with or without BDL (Sham-WT, n = 8; Sham-*Tnfrsf12a* KO, *n* = 8; BDL-WT, *n* = 8; and BDL-*Tnfrsf12a* KO, *n* = 6). *p < *0.05* vs. Sham-WT; $*p* < *0.05* vs. BDL-WT; **C** Representative images of a liver section by immunohistochemical staining showing pro-GSDMD and cleaved-GSDMD in BDL-WT and BDL-*Tnfrsf12a* KO mice (scale bar: 100 µm); **D** Representative images of a liver section by TEM showing features of pyroptosis, including membrane pores, cell swelling, and plasma disruption in the plasma membrane in WT mice and *Tnfrsf12a*-KO mice after BDL (scale bar: 5 µm or 2 µm).

Considering the most recent findings that hepatocyte pyroptosis contributes to cholestatic liver injury [5, 12], we examined the correlation between hepatic TNFRSF12A and pyroptosis in human and murine cholestasis. Western blot analysis showed that levels of hepatic pyroptosis-related indicators NLRP3, cleaved-caspase 1, and cleaved-GSDMD were significantly increased in patients with OC (Fig. 3A), as well as in BDL-WT mice, along with a dramatic elevation of hepatic TNFRSF12A (Fig. 3B), when compared to corresponding normal controls. However, genetic ablation of *Tnfrsf12a* diminished the increases of these pyroptosis-related indicators in BDL mice but did not affect pro-caspase-1 and full-length GSDMD protein expression (Fig. 3B, C). These data suggested that *Tnfrsf12a* deficiency reduced Caspase-1/GSDMD-mediated hepatocyte pyroptosis in cholestasis. To further confirm our observations, transmitted electron microscopy (TEM) was used to examine characteristic morphological changes of hepatocytes. Analysis of the TEM images revealed disruption of the hepatocyte membrane’s integrity, and the cell swelling and membrane pores were evident in hepatocytes in the BDL-WT mice (red arrow). On the contrary, BDL-*Tnfrsf12a* KO mice had relatively normal cell morphology and intact plasma membrane (Fig. 3D). Taken together, our data provided solid evidence that TNFRSF12A deficiency attenuates cholestasis-induced hepatocyte pyroptosis.

### BAs stimulated TNFRSF12A expression and induced pyroptosis in hepatocytes

**Fig. 4:**
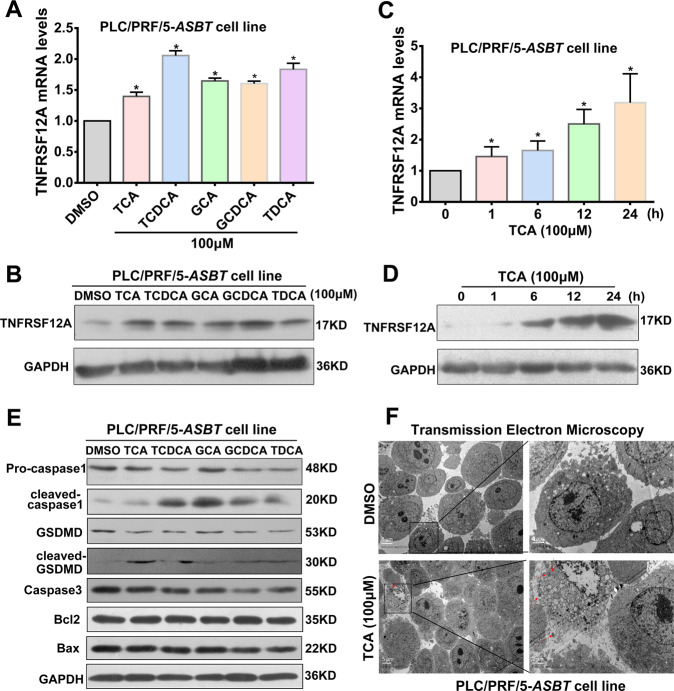
Conjugated BAs induced TNFRSF12A expression and pyroptosis in hepatocytes. **A** TNFRSF12A mRNA transcripts, and (**B**) protein levels in PLC/RPF/5-*ASBT* cells treated with 100 μM TCA, TCDCA, GCA, GCDCA, or TCDCA. *p < 0.05 compared to control (DMSO); Time-dependent effects of TCA on TNFRSF12A (**C**) mRNA and (**D**) protein expression in PLC/RPF/5-*ASBT* cells; (**E**) Western blot analysis of effects of conjugated BAs (TCA, GCA, GCDCA, TDCAC, or TCDCA, 100 μM) on protein expression of pro-Caspase-1, cleaved-Caspase-1, GSDMD, cleaved-GSDMD, caspase-3, Bcl2, and Bax in PLC/RPF/5-*ASBT* cells; **F** Representative images of TEM showing features of pyroptosis, including membrane pores, cell swelling, and large bubbles blowing from the plasma membrane in PLC/RPF/5-*ASBT* cells treated with 100 μM TCA (scale bar: 5 µm).

Next, we investigated whether the accumulated BAs in cholestasis induce TNFRSF12A expression and pyroptosis of hepatocytes. Real-time qPCR and Western blot results demonstrated that conjugated BAs, including TCA, TCDCA, GCA, GCDCA, and TDCA, significantly stimulated TNFRSF12A expression at the mRNA and protein levels in PLC/RPF/5-*ASBT* cell line (Fig. 4A, B). Furthermore, TCA also stimulated TNRSF12A mRNA and protein expression in PLC/RPF/5-*ASBT* cells in a time-dependent manner (Fig. 4C, D). These results were further confirmed in primary mouse hepatocytes (Suppl. Fig. 5A, B). These findings suggested that conjugated BAs induced TNFRSF12A expression in hepatocytes. As shown in Fig. 4E, Western blot analysis revealed that conjugated BAs markedly induced the protein expression of pyroptosis-related indicators cleaved-caspase-1 and cleaved-GSDMD but not apoptotic indicators Caspase-3, Bcl2, and Bax in PLC/RPF/5-*ASBT* cells. Similarly, conjugated BAs also increased cleaved-caspase1, cleaved-GSDMD, cleaved-IL1β and Nlrp3 protein expression in primary mouse hepatocytes (Suppl. Fig. 6). Therefore, conjugated BAs induced pyroptosis but not apoptosis in hepatocytes. Analysis of the TEM images of PLC/PRF/5-*ASBT* cells following TCA treatment exhibited morphological features of hepatocyte pyroptosis, which further confirmed our conclusion (Fig. 4F, denoted by red arrows).

### BA-induced TNFRSF12A initiated hepatocyte pyroptosis via NFκB/Caspase-1/GSDMD signaling

**Fig. 5:**
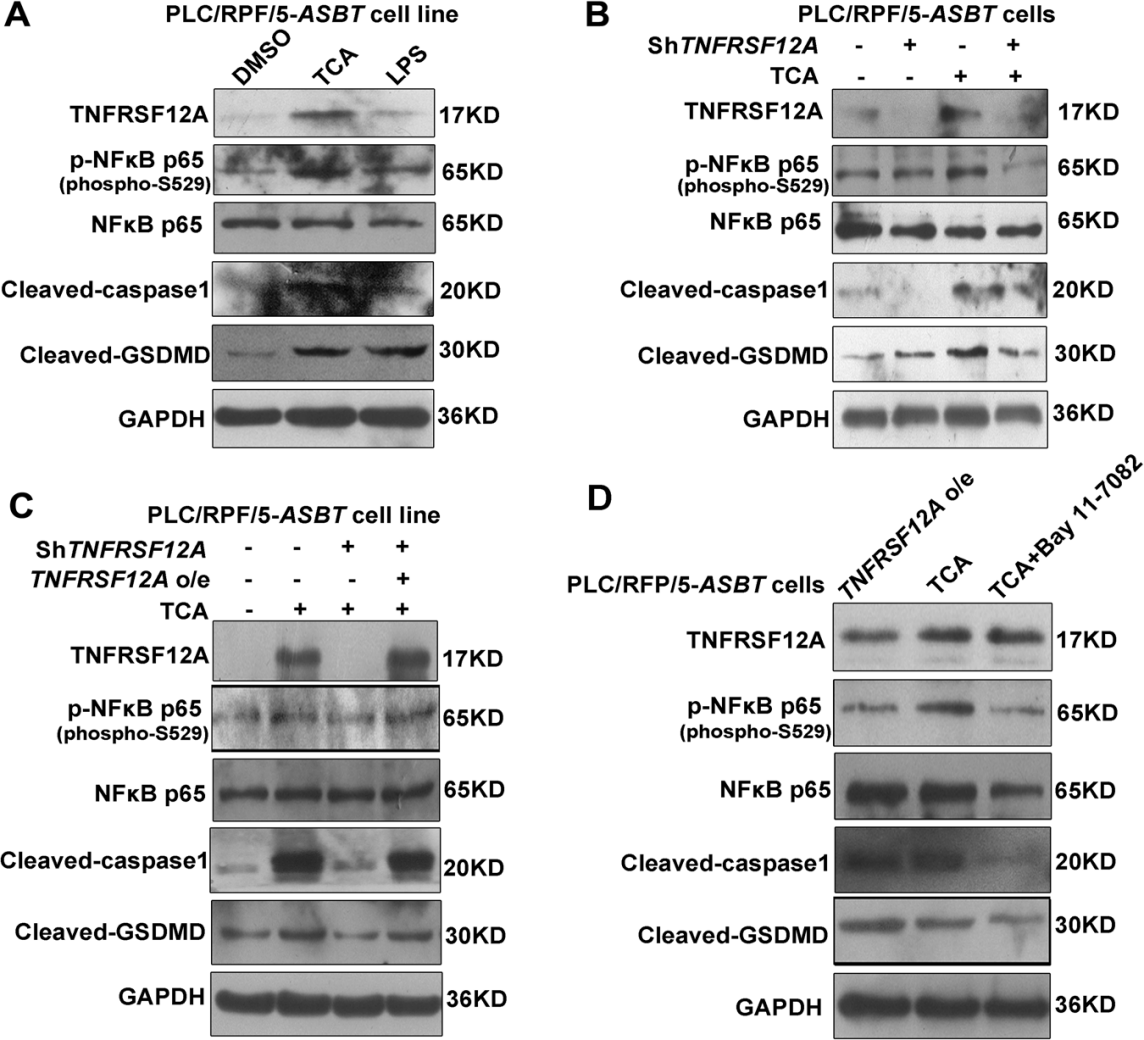
TNFRSF12A initiated hepatocyte pyroptosis via NFκB p65/Caspase-1/GSDMD signaling. Western blot analysis examined proteins of interest in PLC/RPF/5-*ASBT* cells following different treatments. **A** Effects of 100 μM TCA or LPS (100 ng/mL, a positive control) on TNFRSF12A, p-NFκB p65, NFκB p65, Caspase-1, and cleaved-GSDMD; **B** Effects of 100 μM TCA with or without sh-*TNFRSF12A* construct transfection on TNFRSF12A, p-NFκB p65, NFκB p65, cleaved Caspase-1, and cleaved-GSDMD; **C** Effects of 100 μM TCA with or without sh-*TNFRSF12A* or *TNFRSF12A o/e* construct transfection on TNFRSF12A, p-NFκB p65, NFκB p65, cleaved Caspase-1, and cleaved-GSDMD; **D** Effects of 100 μM TCA with or without *TNFRSF12A o/e* construct transfection or Bay 11-7082 treatment on TNFRSF12A, p-NFκB p65, NFκB p65, cleaved Caspase-1, and cleaved-GSDMD.

To gain insight into TNFRSF12A-mediated hepatocyte pyroptosis in cholestasis, we performed mechanistic studies in PLC/RPF/5-*ASBT* cells with TCA or LPS (a positive control) treatment. Western blot analysis showed that TCA and LPS significantly increased the levels of NFκB p65 phosphorylation, cleaved-caspase-1, and cleaved-GSDMD in PLC/RPF/5-*ASBT* cells when compared to their controls (Fig. 5A). Next, we transiently transfected sh-*TNFRSF12A* construct to downregulate the expression of TNFRSF12A. Interestingly, with *TNFRSF12A* knockdown the BA-induced increases in these interest proteins were abolished in PLC/RPF/5-*ASBT* cells (Fig. 5B). However, when the downregulated TNFRSF12A expression in PLC/RPF/5-*ASBT* cells by the sh-*TNFRSF12A* transfection was restored by the *TNFRSF12A o/e* transfection, the levels of NFκB p65 phosphorylation, cleaved-Caspase-1, and cleaved-GSDMD were also recovered (Fig. 5C). These data indicated that TNFRSF12A induced hepatocyte pyroptosis and activated the NFκB signaling pathway. To examine the role of the NFκB signaling pathway in TNFRSF12A-induced hepatocyte pyroptosis by conjugated BAs, PLC/RPF/5-*ASBT* cells were pretreated with BAY 11-7082, a selective inhibitor of the NFκB signaling pathway, before TCA treatment. As shown in Fig. 5D, inhibition of the NFκB signaling pathway did not affect TNFRSF12A protein expression but abrogated increases in cleaved-Caspase-1 and cleaved-GSDMD proteins induced by TCA in PLC/RPF/5-*ASBT* cells. These results indicated that BAs induced TNFSR12A and subsequently induced hepatocyte pyroptosis via the NFκB/Caspase-1/GSDMD signaling pathways.

### Conjugated BAs stimulated infiltrated macrophages to produce TWEAK, thereby enhancing TNFRSF12A-induced hepatocyte pyroptosis

**Fig. 6:**
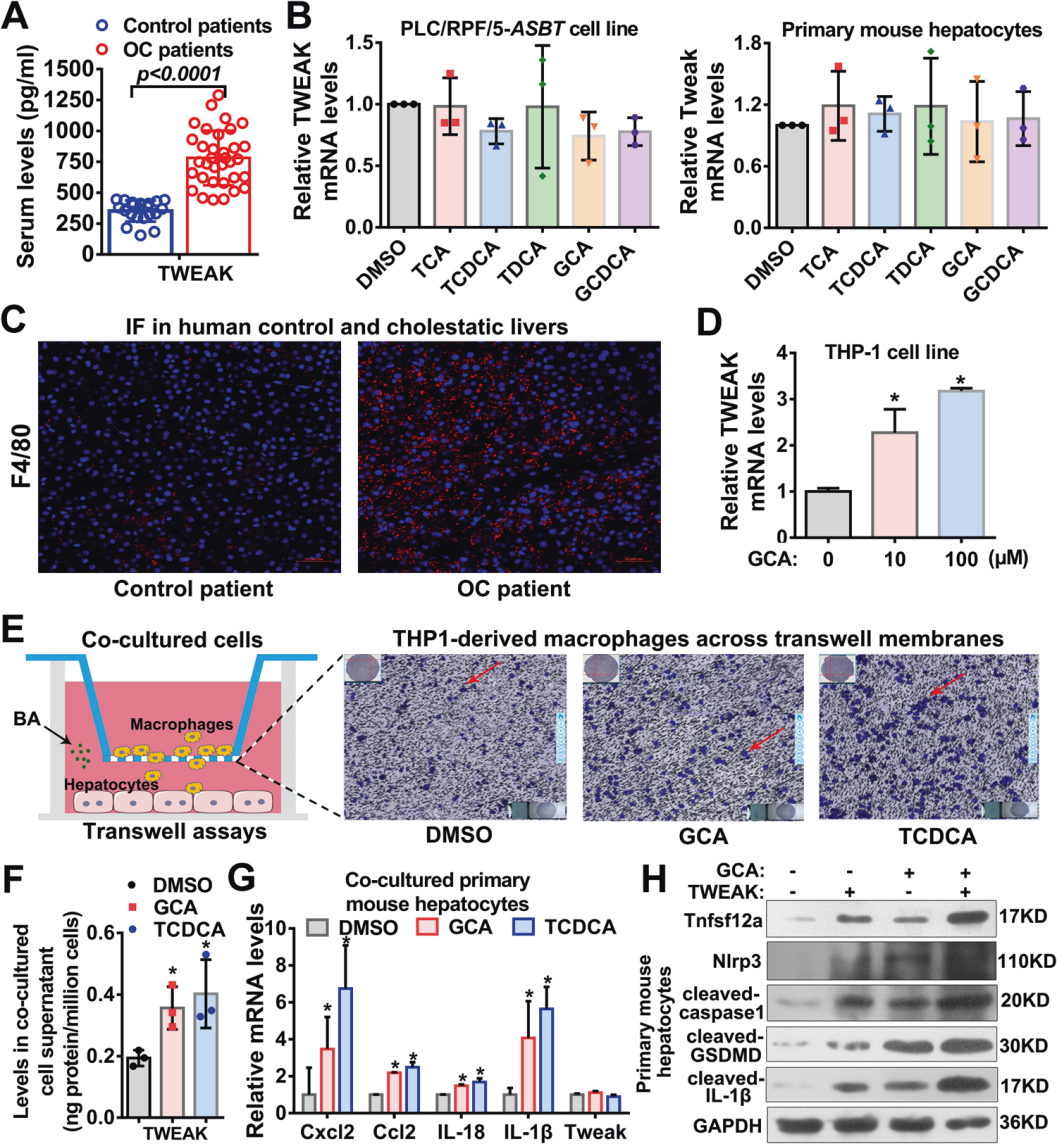
Conjugated BAs stimulated Cxcl2 and Ccl2-mediated macrophage infiltration and subsequent TWEAK production to further enhance TNFRSF12A-induced hepatocyte pyroptosis. **A** Serum TWEAK levels in control patients (*n* = 20) and OC patients (*n* = 34). *p < 0.05 vs. control patients; **B** The levels of TWEAK mRNA transcripts in PLC/RPF/5-*ASBT* cells or primary mouse hepatocytes treated with 100 μM TCA, GCA, GCDCA, TDCAC, and TCDCA; (**C**) Immunofluorescence labeling of F4/80 (a specific marker for macrophages) in a human control liver and an OC liver; **D** The levels of TWEAK mRNA transcripts in THP1 cells treated with 100 μM GCA. *p < 0.05 vs. DMSO control group; **E** A diagram for transwell assays (left) and migration ability of co-cultured THP1-derived macrophages (right) under conjugated BA stimulation; **F** The TWEAK levels in cell supernatant of co-cultured THP-derived macrophages and primary mouse hepatocytes following treatment with DMSO as control, GCA or TCDCA; **G** The levels of Cxcl2, Ccl2, IL-18, IL-1β, and Tweak mRNA transcripts in co-cultured primary mouse hepatocytes following treatment with DMSO as control, GCA or TCDCA. *p < 0.05 vs. DMSO control group; **H** The levels of Tnfrsf12a, Nlrp3, cleaved-Caspase-1, cleaved-GSDMD, and cleaved- IL-1β in primary mouse hepatocytes treated with DMSO as control, GCA, and/or TWEAK. OC, obstructive cholestasis.

Furthermore, we assessed whether and how TWEAK plays a role in TNFRSF12A-induced hepatocyte pyroptosis. As shown in Fig. 6A, serum TWEAK levels were significantly elevated in patients with OC compared to control patients, similar to the observations in human cholestatic livers (Fig. 1A, B). However, conjugated BAs, including TCA, GCA, TCDCA, GCDCA, and TDCA, did not alter TWEAK mRNA expression in both PLC/RPF/5-*ASBT* cells and primary mouse hepatocytes (Fig. 6B). Therefore, the elevated levels of TWEAK were likely attributable to other liver cells. Interestingly, IF labeling revealed that the accumulated hepatic macrophages were remarkably increased in liver tissues of patients with OC compared to control patients (Fig. 6C). Moreover, conjugated BA (GCA) significantly stimulated TWEAK mRNA expression in THP-1 cells (Fig. 6D). These data suggested that conjugated BAs induced the production of TWEAK in macrophages but not hepatocytes.

Next, primary mouse hepatocytes and THP-1-derived macrophages were co-cultured in transwell plates and treated with conjugated BAs (GCA and TCDCA) (Fig. 6E, left). Interestingly, we observed that numerous macrophages migrated across the transwell membranes (Fig. 6E), and the levels of TWEAK were elevated in co-cultured supernatant (Fig. 6F) when co-cultured with primary mouse hepatocytes treated with conjugated BAs (GCA or TCDCA). Furthermore, we also found that treatment with conjugated BAs increased mRNA expression of chemokines (Ccl2 and Cxcl2), IL-18, and IL-1β, but not TWEAK mRNA expression in co-cultured primary hepatocytes (Fig. 6G). These data suggested that BAs induced Ccl2 and Cxcl2 expression in hepatocytes, promoting macrophage infiltration to produce TWEAK.

To further delineate the role of TWEAK in cholestasis, primary mouse hepatocytes were treated with TWEAK and/or conjugated BAs (GCA). As shown in (Fig. 6H), the levels of Tnfrsf12a, Nlrp3, cleaved-Caspase-1, cleaved-GSDMD, and cleaved-IL-1β proteins were higher in the GCA plus TWEAK-treated group compared with either treatment alone, indicating that TWEAK enhanced BA-induced hepatocyte pyroptosis. Altogether, our findings supported the notion that conjugated BAs stimulated Ccl2 and Cxcl2 expression in hepatocytes and subsequently promoted macrophage infiltration to produce TWEAK, leading to further enhance TNFRSF12A-induced hepatocyte pyroptosis.

### Transcription factor c-JUN is involved in the regulation of BAs-induced TNFRSF12A expression in human cholestasis

**Fig. 7:**
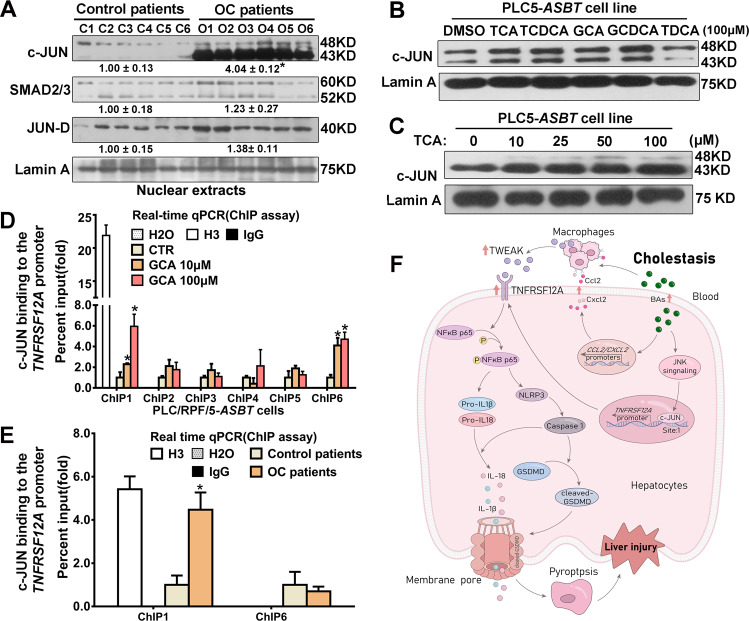
Conjugated BAs increased c-JUN protein expression in the nuclei and the activity of c-JUN binding to the *TNFRSF12A* promoter in PLC/RPF/5-*ASBT* cells and human cholestatic livers. **A** Western blot analysis of protein levels of transcription factors c-JUN, JUN-D, and SMAD2/3 in nuclear extracts from human control and OC livers. *p < 0.05 vs. control patients; **B** Western blot analysis of nuclear expression of transcription factor c-JUN in the PLC/RPF/5-*ASBT* cells treated with 100 μM TCA, TCDCA, GCA, GCDCA, or TDCA. **C** Western blot analysis of nuclear expression of transcription factor c-JUN in PLC/RPF/5-*ASBT* cells treated with 0, 10, 25, 50, 100 μM TCA; (**D**) ChIP assay data showed increased activities of c-JUN binding to the *TNFRSF12A* promoter (ChIP site 1) in a dose-dependent manner in PLC/RPF/5-*ASBT* cells and (**E**) in liver tissues of patients with obstructive cholestasis, compared with control patients. *p < 0.05 vs. their corresponding controls; **F** A schematic diagram illustrating the potential mechanism by which BAs induced TNFRSF12A expression to trigger hepatocyte pyroptosis in cholestatic liver injury.

To gain insight into the regulatory mechanisms of BA-induced TNFRSF12A expression during cholestasis, we first performed in silico analysis of transcription factor binding sites in the promoter region of *TNFRSF12A* (http://jaspar.genereg.net). Numerous putative response elements for c-JUN, JUN-D, and SMAD2/3 in the promoter region of *TNFRSF12A* were identified (Suppl. Fig. 7). Next, we determined the expression of these predicted transcription factors in human cholestatic livers. We found that the levels of hepatic c-JUN protein in nuclei were dramatically increased. In contrast, the levels of nuclear expression of JUN-D and SAMD2/3 were unchanged in liver tissues of patients with OC compared to control patients (Fig. 7A). Furthermore, we also found that conjugated BAs, including TCA, TCDCA, GCA, GCDCA, and TDCA, markedly increased nuclear expression of c-JUN in a dose-dependent manner in PLC/RPF/5-*ASBT* cells (Fig. 7B, C). ChIP assays (real-time qPCR method) demonstrated that the binding activities of c-JUN to the *TNFRSF12A* promoter were significantly increased (ChIP site 1) in a dose-dependent manner in PLC/RPF/5-*ASBT* cells and in liver tissues of patients with OC compared to control patients (Fig. 7D, E). Taken together, these data suggested that conjugated BAs induced the expression of TNFRSF12A in human cholestasis by enhancing the activity of c-Jun binding to the *TNFRSF12A* promoter.

## Discussion

In this study, we report for the first time that hepatic TNFRSF12A expression is remarkably upregulated in patients with OC and PBC, and its elevation is positively correlated with cholestatic liver injury (Fig. 1). Genetic deletion of *Tnfrsf12a* significantly attenuates cholestatic liver injury and pyroptosis in cholestatic mice (Figs. 2, 3 and Tables 1, S5), which is achieved through the following mechanisms (Fig. 7E): (1) The accumulated BAs stimulate TNRFSF12A expression in hepatocytes by increasing the activity of c-JUN binding to the *TNFRSF12A* promoter (Fig. 7); (2) The BA-induced TNRFSF12A initiates hepatocyte pyroptosis by the NFκB/Caspase-1/GSDMD signaling pathway (Figs. 3–5); and (3) BAs stimulate infiltrated macrophages to produce TWEAK, thereby further enhancing TNFRSF12A-initiated hepatocyte pyroptosis during cholestasis (Fig. 6). Therefore, targeting TNFRSF12A could be a new approach for treating cholestasis.

Recent studies demonstrated that hepatic TNFRSF12A was markedly increased in NASH, alcoholic liver disease, chronic hepatitis C, and hepatocellular carcinoma [20, 21]. Interestingly, hepatic Tnfrsf12a is also upregulated in hepatic progenitor cells in BDL- or 0.1%DDC-induced mouse models of cholestasis [22, 23]. However, regulatory mechanisms controlling the hepatocyte Tnfrsf12a gene expression and its functional significance in human cholestasis remain unclear. In this study, we first reported that hepatic TNFRSF12A expression was dramatically increased in human cholestasis, and its elevation was positively associated with cholestatic liver injury (Fig. 1). Knockdown and overexpression studies provided multiple lines of evidence in support of a novel role for TNFRSF12A in the induction of hepatocyte pyroptosis in cholestasis (Figs. 3–5). TWEAK, produced by infiltrated macrophages in cholestatic livers can enhance TNFRSF12A-induced hepatocyte pyroptosis by conjugating BAs (Fig. 6). Han et al. recently also demonstrated that Sestrin2 attenuated cholestatic liver injury by reducing pyroptosis induced by endoplasmic reticulum stress and NLRP3 inflammasome [12]. Therefore, our findings, together with Han et al., identified hepatocyte pyroptosis as a novel cell death mechanism underlying liver injury in patients with cholestasis.

It has been shown that caspase-1 and caspase-11/4/5 cleave full-length GSDMD to release the N-terminal domain as the final pyroptotic executor to punch holes in the cell membrane, resulting in pyroptosis [7–9]. Here, we observed hepatocyte pyroptosis as determined by TEM analysis, as well as the increased levels of pyroptosis-related indicators (cleaved-caspase-1 and cleaved-GSDMD) in cholestatic human and mouse livers (Fig. 3), and the results were consistent with previous reports [11, 12]. Further studies revealed that conjugated BAs induced pyroptosis through increasing cleaved-Caspase-1 and cleaved-GSDMD protein expression in human hepatoma PLC/RPF/5-*ASBT* cells and primary mouse hepatocytes, which was further confirmed by TEM analysis (Fig. 4). It is somewhat different from the previous study [12], which may be attributed to the specificity and sensitivity of Caspase-1 antibody used in primary mouse hepatocytes. Given GSDMD as the common substrate of both pathways, pyroptosis is defined as GSDMD-mediated inflammatory programmed necrosis [6]. Most recently, Susanne et al. also found that pyroptosis was significantly down-regulated in *GSDMD*-KO mice [24], which was in agreement with our findings that cleaved-GSDMD was highly upregulated in cholestasis (Fig. 3). Furthermore, knockdown or knockout of *TNFRSF12A* markedly reduced the levels of NFκB p65 phosphorylation, cleaved-Caspase-1, and cleaved-GSDMD proteins in BA-treated hepatocytes or BDL mouse livers (Fig. 4). Moreover, inhibition of the NFκB signaling pathway also decreased cleaved-Caspase-1 and cleaved-GSDMD protein expression (Fig. 4), indicating that TNFRSF12A induced hepatocyte pyroptosis through activating the NFκB signaling pathway. A recent study reported that inhibition of NFκB signaling by sulfasalazine attenuates cholestatic liver injury in BDL mice [25], in consistent with our observation. Altogether, our findings support the notion that the BA-activated TNFRSF12A-NFκB/Caspase-1/GSDMD signaling pathways play critical roles in aggravating the cholestatic liver injury, which is further enhanced by its ligand TWEAK.

Currently, the regulatory mechanisms controlling hepatic TNFRSF12A expression in human cholestasis remain unknown. Farnesoid X receptor (FXR) has been considered as a key BA-activated nuclear receptor in the regulation of genes involved in biosynthesis, conjugation, and transport of BAs. Its activation has been shown to attenuate cholestatic liver injury [1–3]. Therefore, we examined whether FXR is involved in regulatinghepatic TNFRSF12A expression in cholestasis. Unexpectedly, in silico analysis in this study did not identify any putative FXR/RXR response element within the promoter region of *TNFRSF12A* (Suppl. Fig. 7). Furthermore, we did a literature search, but there were no publications on the interaction between FXR and *TNFRSF12A*. However, our previous studies have demonstrated that conjugated BAs activate hepatic JNK signaling in human cholestasis [26, 27]. JNK-mediated phosphorylation enhances the activity of transcription factor c-JUN, a component of the AP-1 transcription factor to induce transcription [26, 27]. Our recent report also indicates that BA-stressed hepatocytes activate NFAT, which can associate with AP-1 [28]. Indeed, we identified putative response elements for c-JUN in the promoter region of *TNFRSF12A*, and the remarkable increases in nuclear c-JUN protein and the binding activity of c-JUN to the *TNFRSF12A* promoter in human cholestatic livers (Fig. 7). Furthermore, we also found that a variety of conjugated BAs increased nuclear c-JUN protein expression and its binding activity to the *TNFRSF12A* promoter in PLC/RPF/5-*ASBT* cells (Fig. 7A–D). Overall, this study has demonstrated that BAs up-regulate *TNFRSF12A* expression at the transcriptional level in the cholestatic liver through activating the JNK/c-Jun signaling pathway.

Based on these findings in patients with cholestasis, cholestatic mice, and cell cultures, we proposed a new mechanism for cholestatic liver injury (Fig. 7E). Initially, the accumulated intrahepatic BAs during cholestasis activate the JNK signaling pathway [26, 27] to stimulate TNFRSF12A expression at the transcriptional level through increasing the binding activity of c-JUN to the *TNFRSF12A* promoter. Subsequently, the elevation of TNFRSF12A triggers hepatocyte pyroptosis by the NFκB/Caspase-1/GSDMD signaling in cholestasis. Meanwhile, elevated levels of TWEAK produced by infiltrated macrophages in cholestatic livers, can further enhance TNFRSF12A-initiated hepatocyte pyroptosis during cholestasis. However, how BAs stimulate TWEAK production in the infiltrated macrophages and how the NFκB signaling activates NLRP3 expression in hepatocytes remain undetermined. Nevertheless, this is the first report on deciphering the functional significance and regulatory mechanism of TNFRSF12A in human cholestasis.

In summary, our findings have provided the first evidence that hepatic TNFRSF12A expression remarkably increased in human cholestasis and its elevation positively correlated with cholestatic liver injury. Genetic ablation of *Tnfrsf12a* in mice significantly attenuates cholestatic liver injury by inhibiting the BA-induced hepatocyte pyroptosis. Therefore, our findings shed new light on the complicated molecular mechanisms of cholestatic liver injury. Targeting TNFRSF12A or blocking its mediated hepatocyte pyroptosis might be a new therapeutic strategy for treating cholestasis.

## Material and methods

### Ethical approval

Written informed consent was obtained from all patients. This study was approved by the Institutional Ethics Review Board of the First Affiliated Hospital of Army Military Medical University and carried out in compliance with the Declaration of Helsinki (2013) of the World Medical Association.

The protocols involving experimental animals were approved by the Institutional Animal Use and Care Committee of Southwest Hospital, affiliated with the Third Military Medical University.

### Human subjects

Patients with OC and PBC were enrolled at Southwest Hospital, affiliated with the Third Military Medical University (Chongqing, China). OC liver tissues (*n* = 34) were collected from patients undergoing partial hepatectomy to treat pancreatic or periampullary malignancies. Seven patients diagnosed with PBC by elevated serum alkaline phosphatase (ALP) levels and positive anti-mitochondrial antibodies, with autoimmune hepatitis and other liver diseases excluded, had liver biopsies. For comparison, histologically normal liver tissues were taken from surgically resected hepatic or metastatic tumor tissues of 20 adults without cholestasis. Blood samples were collected for subsequent biochemical tests performed by the clinical laboratory at our hospital. The liver tissues from patients undergoing partial hepatectomies were immediately cut into small pieces and fixed in 4% paraformaldehyde or snap-frozen in liquid nitrogen for subsequent analysis. The clinical features and laboratory results of OC and control patients are summarized in Table S1.

### Generation and verification of Tnfrsf12a and Abcb4 knockout (KO) mice

Tnfrsf12a knockout mice (Tnfrsf12a KO) were generated by Genechem Inc (Shanghai, China). In brief, the TALEN vectors targeting exon 2 of Tnfrsf12a were engineered, and plasmids encoding TALEN enzymes were constructed by Golden Gate assembly of the required RVDs into pTAL3 using the Addgene system. TALEN-A and 10 TALEN-B were designed against the sequence 5′-GTCCCCTCCACCCCAC-3′ and 5′-TTCTGTCACCAATCCT-3′, respectively. The coding regions of each plasmid were cloned into the mammalian expression vector, Ptalen_v2_NI, via Bsa I and Bsa I to generate plasmids TALEN-A and TALEN-B. TALEN-mediated Tnfrsf12a F0 mice were screened via the T7E1, and the founder line of four Tnfrsf12a heterozygous mutant mice (F0) was crossed to and maintained in the C57BL/6 background. The genotype of F1 mice was examined by PCR and confirmed by DNA sequencing. Heterozygous breeding pairs generated Tnfrsf12a homozygous mice (Tnfrsf12a KO) and wild-type (WT) littermates.

Abcb4 KO mice (Abcb4-KO, C57BL/6J background) were developed by Shanghai Model Organisms Center, Inc (Shanghai, China), as we previously reported [26]. Briefly, a deletion of 411 bp in exon 3 of the Abcb4 gene resulted in a frameshift mutation and gene inactivation in Abcb4 KO mice.

### Experimental animals

All C57BL/6 J mice were housed in 12 h light/dark cycles, with free access to food and water. For the bile duct ligation (BDL) experiments, 8-week-old WT mice and *Tnfrsf12a* KO mice were randomly divided into four groups to receive BDL or sham operation for seven days: sham operation group (*n* = 8 for each genotype) and BDL group (*n* = 8 for WT mice and *n* = 6 for *Tnfrsf12a* KO mice). For the 14-day 0.1%DDC feeding experiments, 8-week-old WT and *Tnfrsf12a* KO mice were each divided into four groups: chow diet group (*n* = 5 for each genotype) and 0.1%DDC diet group (*n* = 4 for each genotype). For the *Abcb4* deficiency-induced mouse model of cholestasis (progressive familial intrahepatic cholestasis type 3), WT mice (*n* = 11) and *Abcb4*-KO mice (*n* = 10) were divided into two groups. Blood samples were collected and immediately stored at −80°C until further use. The fresh mouse liver tissues were perfused with phosphate-buffered saline (PBS) to flush out blood and then were cut into small pieces and rapidly frozen in liquid nitrogen for subsequent analysis.

### Generation of TNFRSF12A shRNA knockdown and overexpression constructs

Knockdown (KD) of *TNFRSF12A* was performed by transfection of shRNA (Shanghai Genechem). *TNFRSF12A* overexpression construct (*TNFRSF12A o/e*) was purchased from GeneCopoeia (EX-V0431-M98, Rockville, MD, USA). Transfection of Sh-*TNFRSF12A* or *TNFRSF12A* o/e was performed per the manufacturer’s protocol.

### Cell culture and treatments

The human hepatoma PLC/PRF/5 and THP-1 cell lines (ATCC, Manassas, VA, USA) and PLC/PRF/5-*ASBT* cells were used as described previously [29]. Cells were cultured in DMEM or RPMI-1640 medium (Gibco) containing 10% fetal bovine serum (FBS, complete medium, HyClone) at 37 °C, 5% CO_2_. Before stimulation with LPS (100 ng/ml), TCA (100 μM), or DMSO, PLC/RPF/5-*ASBT* cells were pretreated with 10 μM BAY 11-7082, a selective inhibitor of NFκB signaling, for 30 min and treated with 100 μM glycocholate acid (GCA), glycocholic acid (GCDCA), taurocholic acid (TCA), taurochenodeoxycholic acid (TCDCA) or taurohyodeoxycholic acid (TDCA) for 24 h. THP-1 cells or THP-1-derived macrophages induced by phorbol 12-myristate 13-acetate (PMA) (MedChemExpress, USA; Cat# 16561-29-8) [30] were treated with conjugated BAs or used for transwell assays. The harvested cells were lysed for subsequent real-time qPCR or Western blot analysis.

### Preparation and treatment of primary mouse hepatocytes

Primary mouse hepatocytes were isolated from 10- and 20-week-old mice using collagenase perfusion as previously described [26]. Fresh primary mouse hepatocytes were cultured or co-cultured in 5% FBS–Williams’ Medium E and subsequently treated with 100 μM conjugated BAs or 100 ng/ml TWEAK (Peprotech, NJ, USA) for 12 or 24 h. Finally, whole cell lysates were collected as described previously [26, 29] for subsequent real-time qPCR and Western blot analysis.

### THP-1-derived macrophages and primary mouse hepatocytes co-culture and transwell assays

Transwell migration assays were performed in 6-well Transwell plates (Permeable Supports 24-mm insert, 3.0-μm polycarbonate membrane provided by Costar) as described previously [31]. Briefly, 2.5 ml/well of 24-hour cell culture medium was placed for primary mouse hepatocytes treated with or without 100 µM GCA or TCDCA in a 6-well plate, and 1 ml of 0.4 × 106 THP-1-derived macrophages/ml were loaded into the inner chamber and incubated at 37 °C for 6 h. After incubation, the inner chamber was removed, and migrated cells were fixed with 4% paraformaldehyde and counted under a microscope. The number of migrated THP-1-derived macrophages was normalized to the initial number of hepatocytes from which the culture medium was collected.

### Transmission electron microscopy

Liver tissues were fixed with 4% paraformaldehyde (PFA), dehydrated by 30% sucrose, and Paraffin tissue sections were prepared at room temperature (RT). Liver sections of approximately 50 nm thickness were cut using an ultramicrotome with a diamond knife (Thermo, HM325, USA), and samples were examined by TEM to detect pores on the cytomembrane.

### Quantitative real-time polymerase chain reaction (qRT-PCR)

Total RNA was extracted from tissues or cultured cells using TRIzol^®^ reagent (Invitrogen, San Diego, CA) per the manufacturer’s instructions previously described [29, 24, 27, 31, 32]. TaqMan probes and specific primers used in the qRT-PCR analysis are listed in Table S2.

### Western blot analysis

Whole-cell lysates and nuclear extracts for Western blot analysis were prepared as described previously [29, 27, 31, 32]. The detailed information on the primary antibodies used in Western blot analysis is shown in Table S3.

### Chromatin immunoprecipitation (ChIP) assay

ChIP assay was performed using a ChIP assay kit (Millipore, Bedford, MA) per the manufacturer’s protocol as previously described [26, 29]. In brief, tissue sonication was carried out to prepare extracts, and then primary antibodies of c-JUN (Abcam, UK) and IgG (Abcam, UK) were used for ChIP assay (Table S3). DNA-binding proteins were isolated and co-purified with genomic DNA as cross-linked DNA-protein complexes, and purified DNA was subjected to qPCR with SYBR green fluorescent dye (Invitrogen, Carlsbad, CA, USA) (Table S4).

### Liver histology

Liver sections were stained with hematoxylin and eosin (H&E) as previously described [26, 29]. Liver histological assessments were performed by expert pathologists who were blinded to patient clinical data.

### Examination of serum TWEAK levels in OC patients

Human serum samples or co-cultured cell supernatant were examined for serum TWEAK levels using a TWEAK ELISA Kit (CAT#YJ712493, Shanghai Enzyme-linked Biotechnology Co., Ltd., Shanghai, China) per the manufacturer’s instructions.

### Statistical analysis

Statistical analysis was conducted using Graph Pad Prism (version 6; Graph Pad Software Inc, USA) and IBM SPSS Statistics version 26.0 (IBM Corporation, USA). Data are expressed as mean ± standard deviation (SD) or standard error of the mean (SEM). Nonparametric data were compared using the Kruskal-Wallis test followed by Dunn’s post hoc test. A comparison of the two groups was analyzed using Student´s t-test. The significance level was set at *p* < 0.05 for all comparisons.

## Supplementary information

Hepatic TNFRSF12A promotes bile acid-induced hepatocyte pyroptosis through NFκB/Caspase-1/GSDMD signaling in cholestasis

Original Data File

## Data Availability

All data that support the findings in this study are available from the corresponding author upon reasonable request.
