# Supplementary material for: Hepatic TNFRSF12A promotes bile acid-induced hepatocyte pyroptosis through NFκB/Caspase-1/GSDMD signaling in cholestasis
Source: Cell Death Discov. 2023 Jan 23;9:26. doi: 10.1038/s41420-023-01326-z (PMC9871041; doi:10.1038/s41420-023-01326-z)
Supplement: Supplementary file 1 — Hepatic TNFRSF12A promotes bile acid-induced hepatocyte pyroptosis through NFκB/Caspase-1/GSDMD signaling in cholestasis [file 41420_2023_1326_MOESM1_ESM.docx]

***Supporting documents***

**Hepatic TNFRSF12A promotes bile acid-induced hepatocyte pyroptosis through NFκB/Caspase1/GSDMD signaling in cholestasis**

Min Liao^1, 2, 3^*, Junwei Liao^3, 4^*, Jiaquan Qu^1, 2, 3, 5^*, Pang Shi^1, 2, 3^*, Ying Cheng^1, 2, 3^, Qiong Pan^1, 2, 3^, Nan Zhao^1, 2, 3^, Xiaoxun Zhang^1, 2, 3^, Liangjun Zhang^1, 2, 3^, Ya Tan^1, 2, 3^, Qiao Li^1, 2, 3^, Jin-Fei Zhu^1, 6^, Jianwei Li^7^, Chengcheng Zhang^7^, Shi-Ying Cai^8^, Jin Chai^1, 2, 3#^

^1^Department of Gastroenterology, ^2^Institute of Digestive Diseases of PLA, ^3^Center for Cholestatic Liver Diseases and Center for Metabolic-Associated Fatty Liver Diseases, The First Affiliated Hospital (Southwest Hospital), Third Military Medical University (Army Medical University), Chongqing 400038, China; E-mail: jin.chai@cldcsw.org

^4^Central South University School of Sciences, Changsha, Hunan 410083, China;

^5^Department of Medical Imaging Technology, Medical College of Jishou University, Jishou, Hunan 416000, China;

^6^Queen Mary School, Nanchang University, Nanchang, Jiangxi 330031, China;

^7^Institute of Hepatobiliary Surgery, Southwest Hospital, Third Military Medical University, Chongqing 400038, China;

^8^Department of Internal Medicine and Liver Center, Yale University School of Medicine, New Haven, CT 06520, USA.

*These authors contributed equally to this study and shared the first authorship.

^#^**Correspondence author:** Jin Chai, M.D., Ph.D.

Professor of Gastroenterology and Hepatology

Chongqing University School of Medicine and Third Military Medical University (Army Medical University)

Department of Gastroenterology, Institute of Digestive Diseases of PLA, Center for Metabolic Associated Fatty Liver Diseases and Center for Cholestatic Liver Diseases, The First Affiliated Hospital (Southwest Hospital), Third Military Medical University (Army Medical University) Chongqing, 400038, China

Tel: 86-23-68765331; Fax: 86-23-65410853

E-mail: [jin.chai@cldcsw.org](mailto:jin.chai@cldcsw.org);

**Grants Support**

This work was supported by grants from the National Natural Science Foundation of China (81922012), the Outstanding Youth Foundation of Chongqing (cstc2021jcyj-jqX0005), the Project of Chongqing Universities Innovation Research / Outstanding Medical Research Group (2021cqspt01 and 4246ZO1), and the Science Foundation of Southwest Hospital and Army Medical University (XZ-2019-505-001 and XZ-2019-505-069), China.

**Conflicts of Interest**

The authors disclose no conflicts.

**Supplementary Figures**


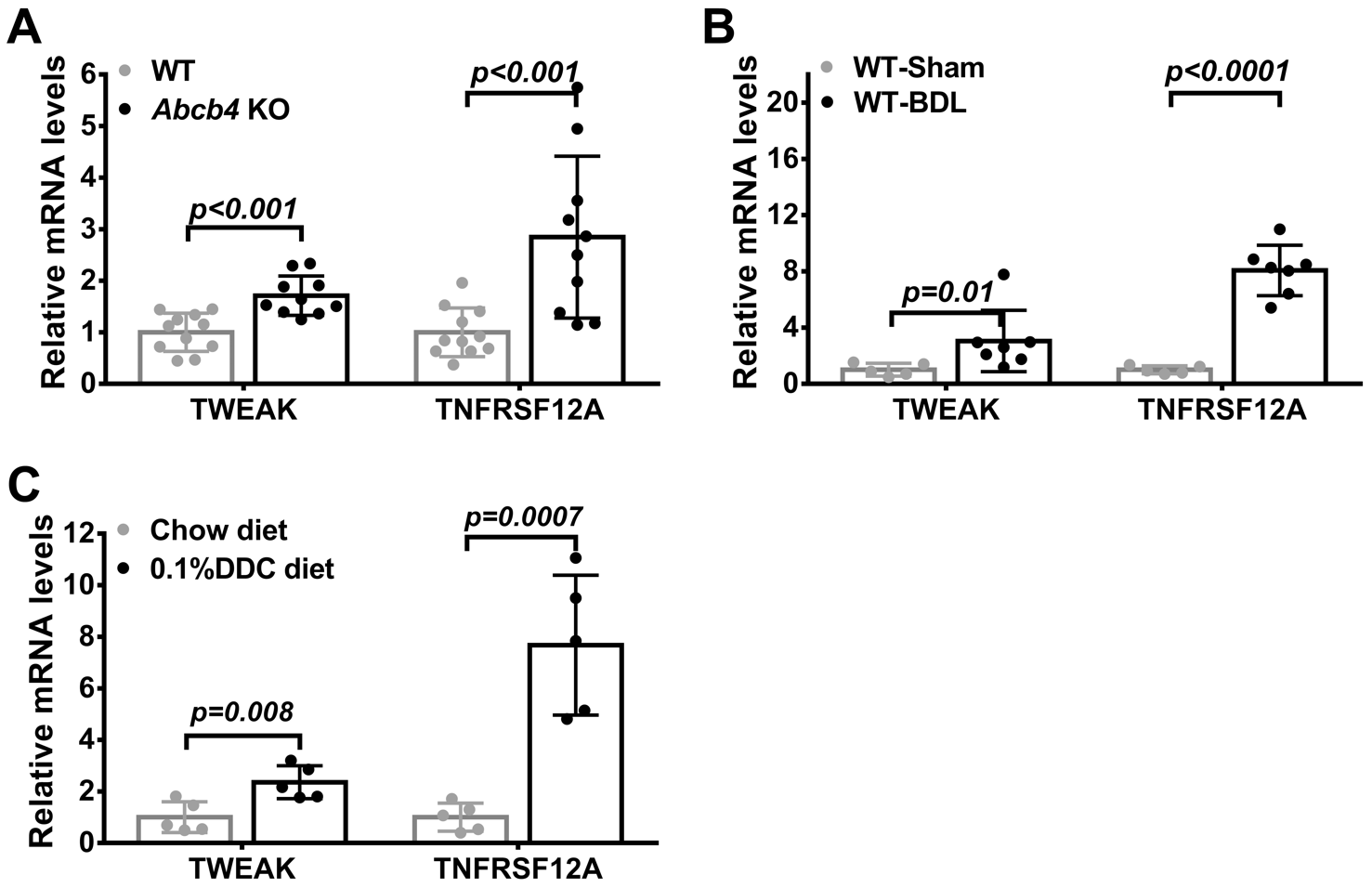


**Suppl. Figure 1. Relative mRNA levels of Tweak and Tnfrsf12a in mouse models of cholestasis induced by BDL, 0.1%DDC, and *Abcb4* knockout.** BDL, bile-duct ligation; WT, wild-type; KO, knockout.


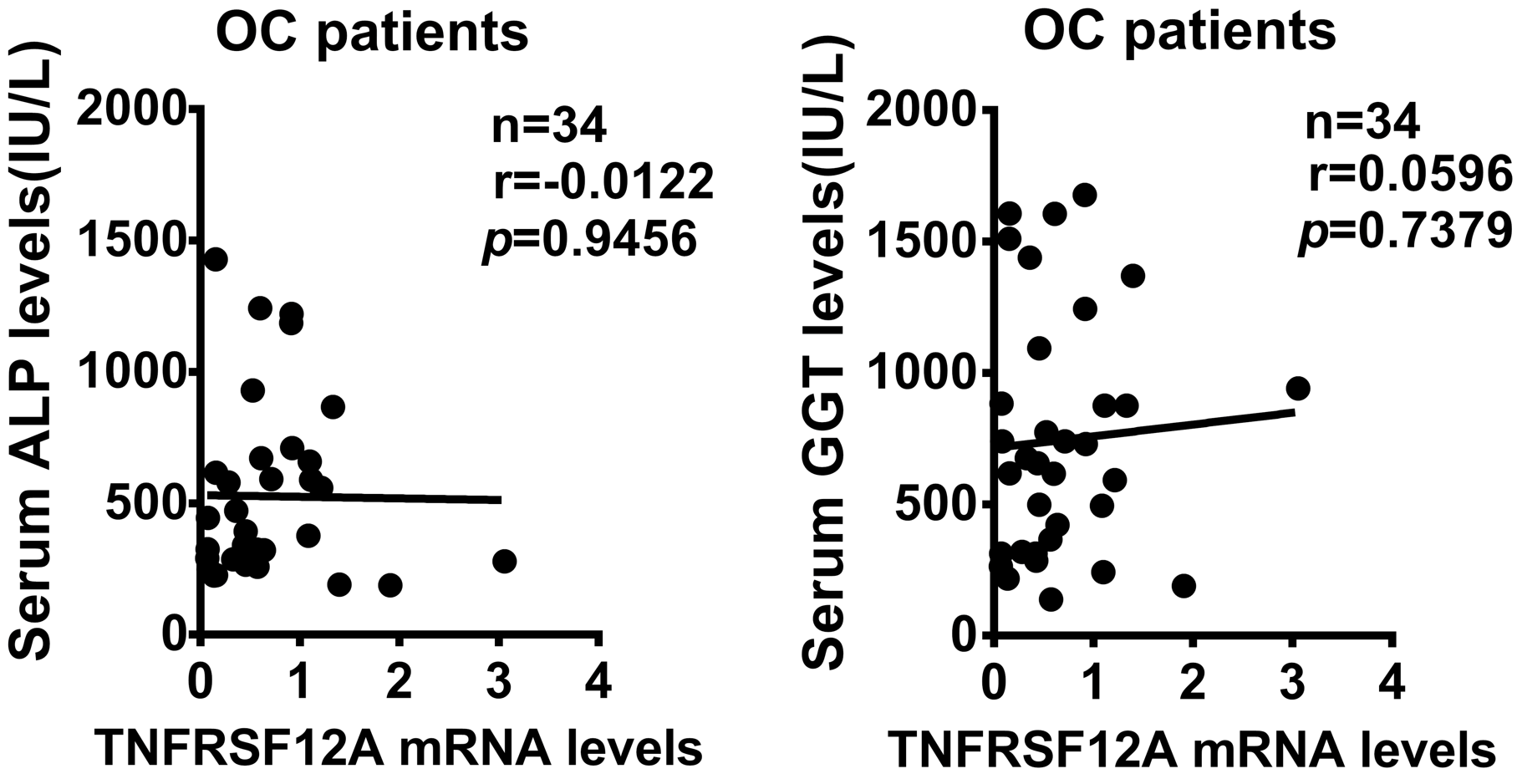


**Suppl. Figure 2. Correlation between hepatic TNFSF12A mRNA levels and serum ALP or GGT levels.** ALP, alkaline phosphatase; GGT, gamma-glutamyl transferase; OC, obstructive cholestasis.


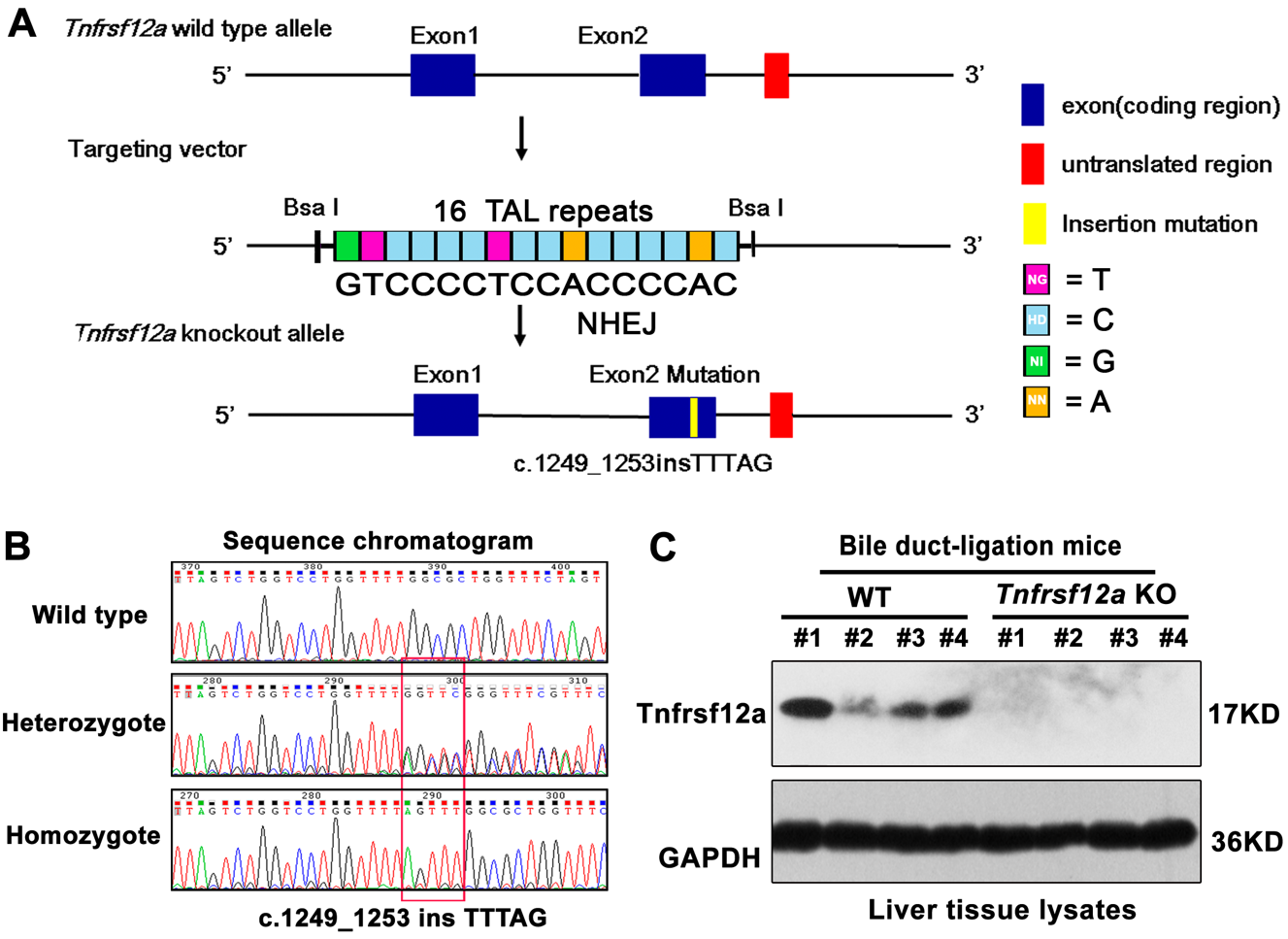


**Suppl. Figure 3. Generation and characterization of *Tnfrsf12a* KO mice.** (A) Schematic diagram of *Tnfrsf12a* KO mice. (B) Sequencing analysis of WT, heterozygous and homozygous *Tnfrsf12a* KO mice. (C) Western blot analysis of Tnfrsf12a expression in WT and Tnfrsf12a KO mice liver tissues. KO, knockout; WT, wild-type.


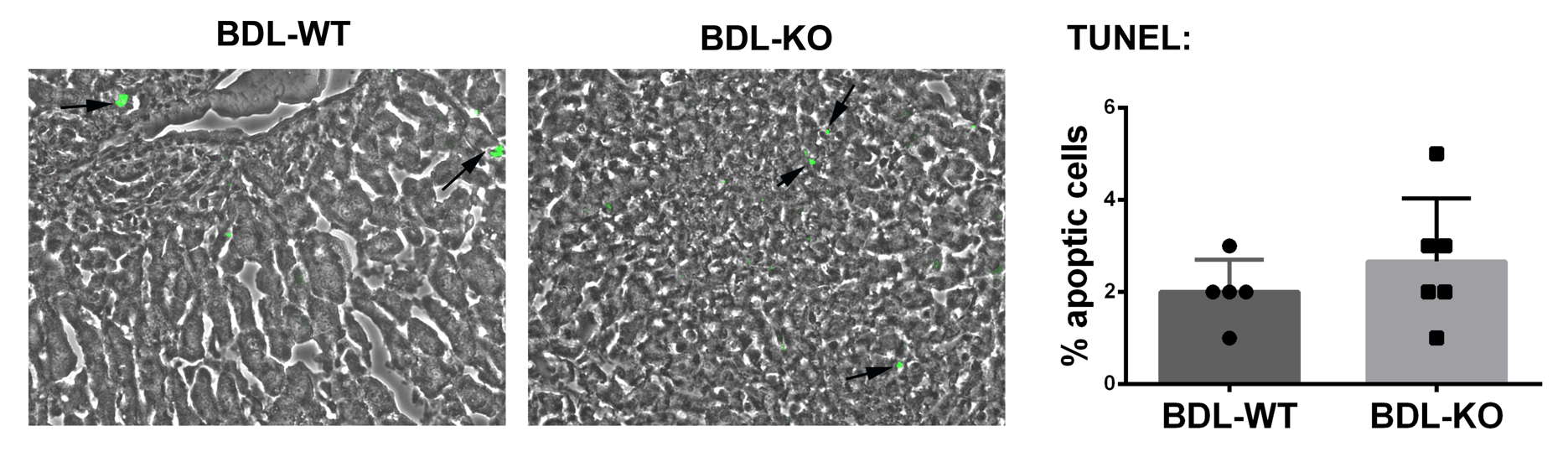


**Suppl. Figure 4. Assessments of hepatic apoptosis in WT and *Tnfrsf12a* KO mice after BDL.** BDL, bile-duct ligation; KO, knockout; WT, wild-type.


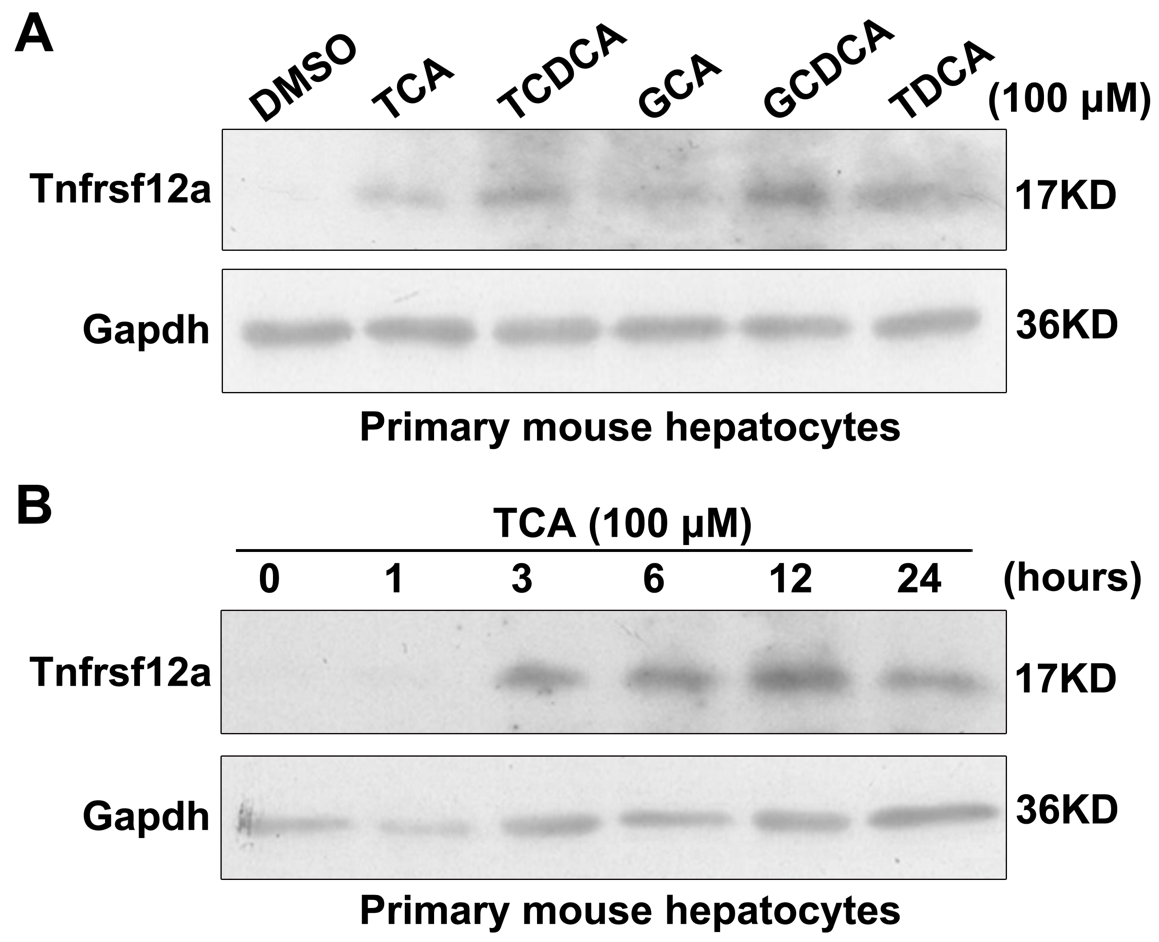


**Suppl. Figure 5. Conjugated BAs induced Tnfrsf12a protein expression in primary mouse hepatocytes.** (A) Conjugated BAs stimulated Tnfrsf12a protein expression; (B) TCA induced Tnfrsf12a expression in a time-dependent manner.


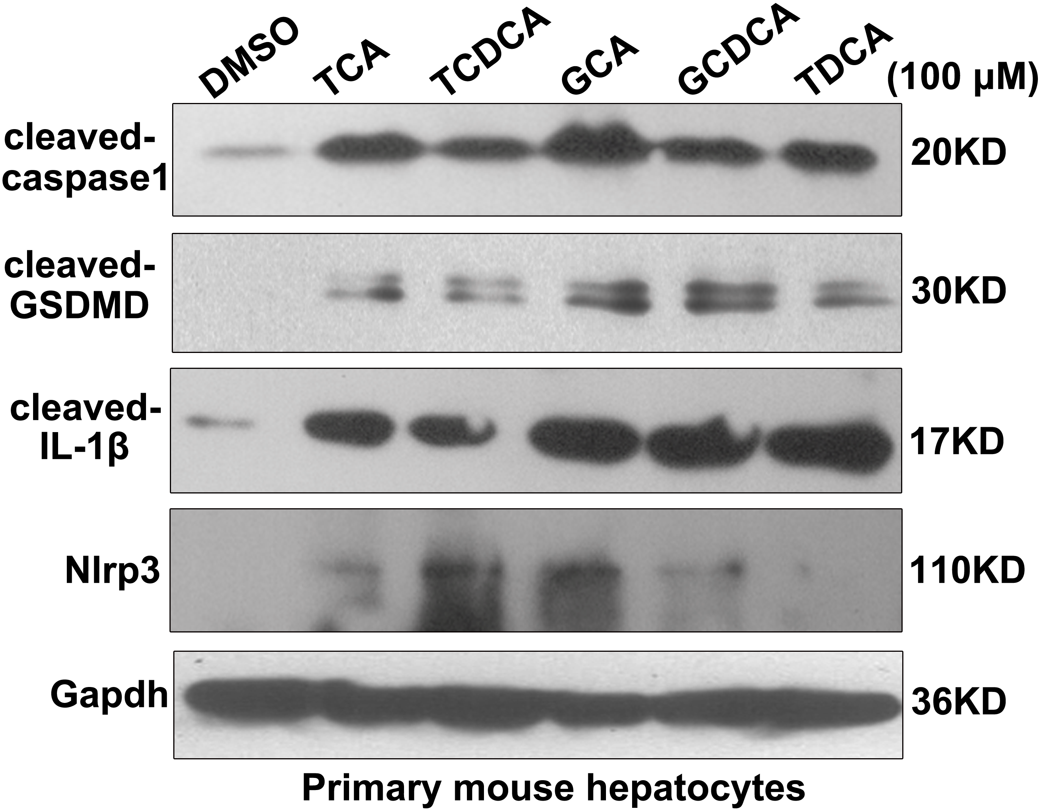


**Suppl. Figure 6. Conjugated BAs induced the levels of cleaved-caspase1, cleaved-GSDMD, cleaved-IL1β and Nlrp3 proteins in primary mouse hepatocytes.**


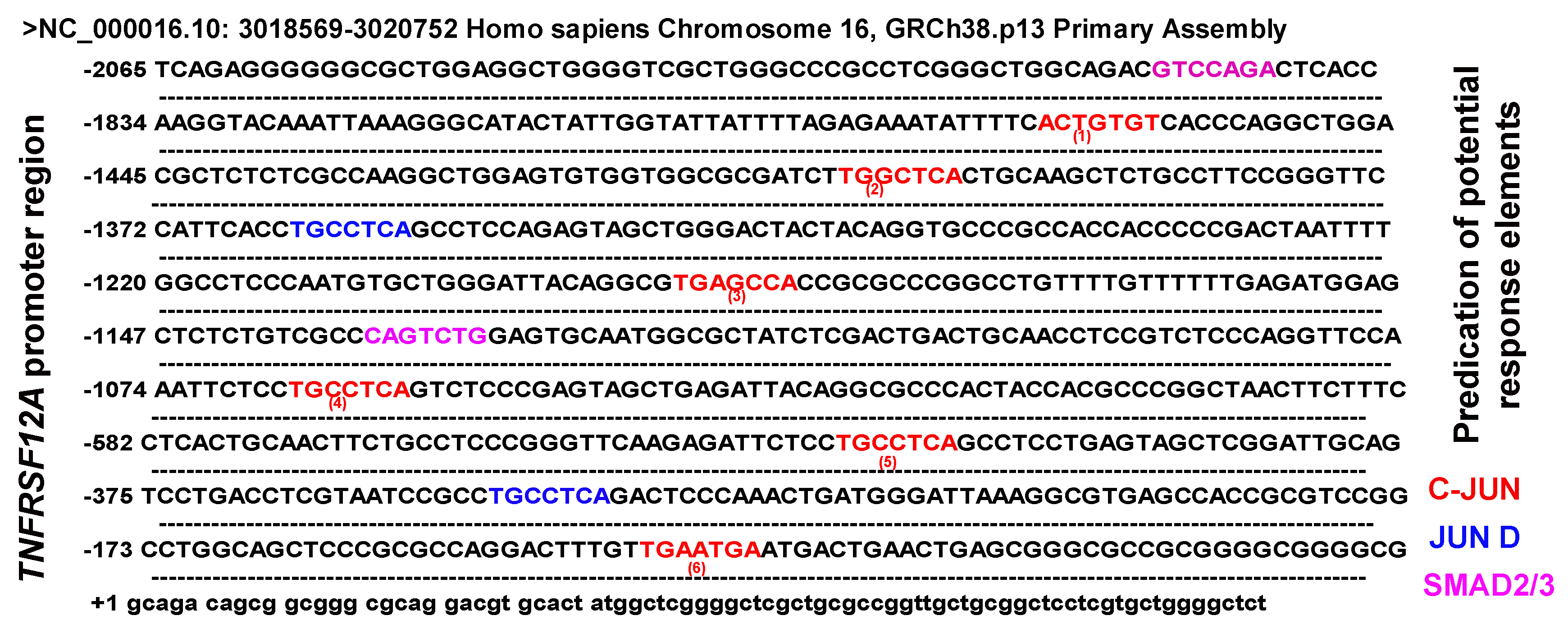


**Suppl. Figure 7. Schematic presentation of the promoter region of *TNFRSF12A***

**Supplementary Tables**

**Table S1 Serum biochemistry parameters in obstructive cholestatic patients and control patients**

| **Liver function tests** | **­ Control patients**  **(n=20)** | **Obstructive cholestatic patients**  **(n=34)** |
| --- | --- | --- |
| **ALT (IU/L)** | 45.28 ± 8.18 | 142.52 ±17.24* |
| **AST (IU/L)** | 42.28 ± 6.04 | 134.96 ± 16­­.46* |
| **ALP (IU/L)** | 99.57 ± 10.39 | 526.68 ± 57.62* |
| **GGT (IU/L)** | 90.60 ± 24.89 | 744.79 ± 78.53* |
| **TBIL (μmol/L)** | 26.92 ± 8.28 | 223.66 ± 34.71* |
| **DBIL (μmol/L)** | 10.26 ±4.98 | 109.22 ± 15.29* |
| **TBA (μmol/L)** | 14.24 ± 10.05 | 98.05 ± 18.26 * |
| **IBIL (μmol/L)** | ­­16.66 ± 3.49 | 114.43 ± 20.91* |

Note: Values are means±SD. *^*^p<0.001* vs control patients. ALT, alanine aminotransferase; AST, aspartate aminotransferase; ALP, alkaline phosphatase; GGT, gamma-glutamyl transferase; TBIL, total bilirubin; DBIL, direct bilirubin; TBA, total bile salts; IBIL, indirect bilirubin.

**Table S2 Real-time qPCR probes (TaqMan) or primers**

| Gene | Sequence (5^/^→3^/^) | Species/Source |
| --- | --- | --- |
| GAPDH | Proprietary to ABI | Human/Hs02758991_gl |
| TNFRSF12A | Proprietary to ABI | Human/Hs00959047_ml |
| TWEAK | Proprietary to ABI | Human/Hs00387540_ml |
| Gapdh | Proprietary to ABI | Mouse/Mm99999915_g1 |
| Tnfrsf12a | Proprietary to ABI | Mouse/Mm01302476_g1 |
| Tweak | Proprietary to ABI | Mouse/Mm02583406-s1 |
| Ccl2 | Proprietary to ABI | Mouse/Mm00441242_m1 |
| IL-18 | Proprietary to ABI | Mouse/Mm00434225_m1 |
| Cxcl2 | Forward: 5'-aggcatctgcttcggggactctggc-3' Reverse: 5'-gcaaactcagccacaggggcgaagg-3' | Mouse/Primers (SYBR) |
| IL-1β | Forward:5'-gaaatgccaccttttgacagtg-3'  Reverse: 5'-tggatgctctcatcaggacag-3' | Mouse/Primers (SYBR) |

**Table S3 Antibodies used in Western blot, chromatin co-immunoprecipitation, and** **immunohistochemistry**

| Protein | Host | Company/Catalog | Antibody dilution |
| --- | --- | --- | --- |
| GAPDH | Rabbit | Proteintech, Chicago, IL/10494-1-AP | WB 1:5000 |
| TNFRSF12A/TWEAKER | Rabbit | Abcam, Cambridge, ab109365 | WB 1:1000; |
| c-JUN | Rabbit | Cell signaling, Boston, #9165 | WB 1: 1000; ChIP 2μg per sample |
| JUN-D | Mouse | Santa Cruz,Dallas, CA/sc-271938 | WB 1:500 |
| SMAD2/3 | Rabbit | ABclonal,Wu Han, A18674 | WB 1:1000 |
| TWEAK | Rabbit | Abcam, Cambridge, ab37170 | WB 1:1000 |
| NLRP3 | Rabbit | Cell signaling, Boston, #15101S | WB 1:1000 |
| Pro-caspase1 | Rabbit | Cell signaling, Boston,24232T | WB 1:1000 |
| cleaved-caspase1 | Rabbit | Cell signaling, Boston,89332T | WB 1:1000 |
| GSDMD | Rabbit | Cell signaling, Boston,93709T | WB 1:1000 |
| cleaved-GSDMD | Rabbit | Cell signaling, Boston,36425T | WB 1:1000 |
| p-NFκB p65 (S529) | Rabbit | Abcam, Cambridge, MA/ab109458 | WB 1:2000 |
| NFκB p65 | Rabbit | Abcam, Cambridge, MA/ab7970 | WB 1:2000 |
| Caspase 3 | Rabbit | Proteintech, Chicago, 19677-1-AP | WB 1:1000 |
| Bcl2 | Rabbit | Proteintech, Chicago, 12789-1-AP | WB 1:2000 |
| Bax | Rabbit | Proteintech, Chicago, 50599-2-Ig | WB 1:5000 |
| F4/80 | Rabbit | Cell signaling, Boston,70076 | WB 1:1000 |
| GAPDH | Rabbit | Proteintech, Chicago, IL/10494-1-AP | WB 1:5000 |

**Table S4 Real-time qPCR primers for chromatin co-immunoprecipitation (ChIP) assays**

| **ChIP** | **Primer pairs** | **Products(bp)** |
| --- | --- | --- |
| c-JUN ChIP1(-405) | Forward: 5’-AGCAGCGCGGTGACATGGGGAACT-3’ | 251bp |
|  | Reverse: 5’-AGGAGGCTGAGGCGGGAGGAT-3’ |  |
| c-JUN ChIP2 (-778) | Forward: 5’-CGCGCCCGGCCAGATAATA-3’ | 165bp |
|  | Reverse: 5’-TGAGGCAGGTGAATGGCGTGAAC-3’ |  |
| c-JUN ChIP3 (-994) | Forward: 5’-GCCTTCCGGGTTCACGCCATTCAC-3’ | 300bp |
|  | Reverse: 5’-ACGGAGGTTGCAGTCAGTCG-3’ |  |
| c-JUN ChIP4 (-1119) | Forward: 5’-AGTGCAATGGCGCTATCTCG-3’ | 164bp |
|  | Reverse: 5’-AACTTGGTGAACCCCCGTCTTTAC-3’ |  |
| c-JUN ChIP5 (-1697) | Forward: 5’-GATCTCGGCTCACTGCAACTTCTG-3’ | 221bp |
|  | Reverse: 5’-GCTCACGCCTTTAATCCCATCA-3’ |  |
| c-JUN ChIP6 (-2040) | Forward: 5’-CGCGTCCGGCCGTTCGTGT-3’ | 169bp |
|  | Reverse: 5’-GGCGCCCGCTCAGTTCAGTCATTC-3’ |  |
| ChIP for positive control (GAPDH) | Reverse: 5’-TGAGGCAGGTGAATGGCGTGAAC-3’ | 166bp |
|  | Reverse: 5’-TCGAACAGGAGGAGCAGAGAGCGA-3’ |  |

**Table S5** **Serum biochemistry in** **mice with 0.1%DDC diet for 14 days**

|  | | | **Chow diet, 14 days** | | |  | |  | | **0.1%DDC diet, 14 days** | | | | | |
| --- | --- | --- | --- | --- | --- | --- | --- | --- | --- | --- | --- | --- | --- | --- | --- |
| **Liver function tests** | | **WT**  **(n=5)** | | ***Tnfrsf12a* KO (n=5)** |  | |  | | **WT**  **(n=4)** | | | ***Tnfrsf12a* KO**  **(n=4)** |  | |  |
| **ALT (IU/L)** | 40.72±6.91 | | 35.68±11.63 | 652.20±142.59*^#^ | | | | | | 403.00±179.24*^#^ | | |  |  |  |
| **AST (IU/L)** | 140.80±43.59 | | 131.92±20.79 | 675.20±261.38*^#^ | | | | | | 285.10±132.55*^#&^ | | |  |  |  |
| **ALP (IU/L)** | 107.20±19.06 | | 88.00±14.42 | 1307.00±212.35*^#^ | | | | | | 471.00±159.55*^#&^ | | |  |  |  |
| **TBA (μmol/L)** | 2.26±0.58 | | 2.95±1.28 | 256.93±124.16*^#^ | | | | | | 64.41±105.13*^#^ | | |  |  |  |
| **TBIL (μmol/L)** | 0.08±1.73 | | 0.00±2.17 | 142.30±112.64*^#^ | | | | | | 31.60±28.44*^#^ | | |  |  |  |

**Notes:** Values are means±SD. ******P<*0.05 VS Chow diet-WT mice; **#***P<*0.05 VS Chow diet-*Tnfrsf12a* KO mice; **&** *P<*0.05 VS 0.1%DDC diet-WT mice. KO, Knock out; ALT, alanine aminotransferase; AST, aspartate aminotransferase; ALP, alkaline phosphatase; TBA, total bile salts; TBIL, total bilirubin.
