# Supplementary material for: Hepatic TNFRSF12A promotes bile acid-induced hepatocyte pyroptosis through NFκB/Caspase-1/GSDMD signaling in cholestasis
Source: Cell Death Discov. 2023 Jan 23;9:26. doi: 10.1038/s41420-023-01326-z (PMC9871041; doi:10.1038/s41420-023-01326-z)

Fig 1C

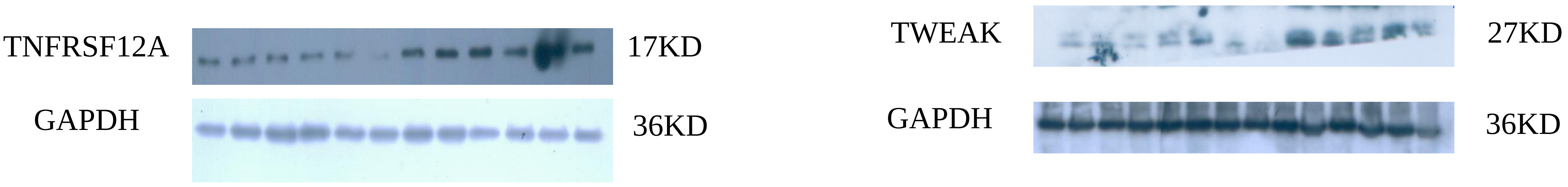
TNFRSF12A
17KD
GAPDH
36KD
TWEAK
27KD
GAPDH
36KD

Fig 3A

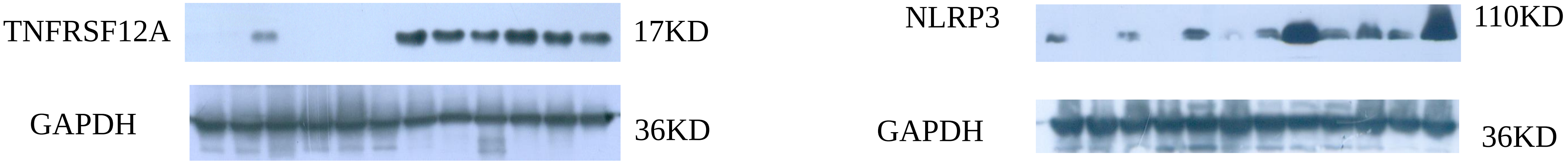
TNFRSF12A
17KD
GAPDH
36KD
NLRP3
110KD
GAPDH
36KD

Fig 3B

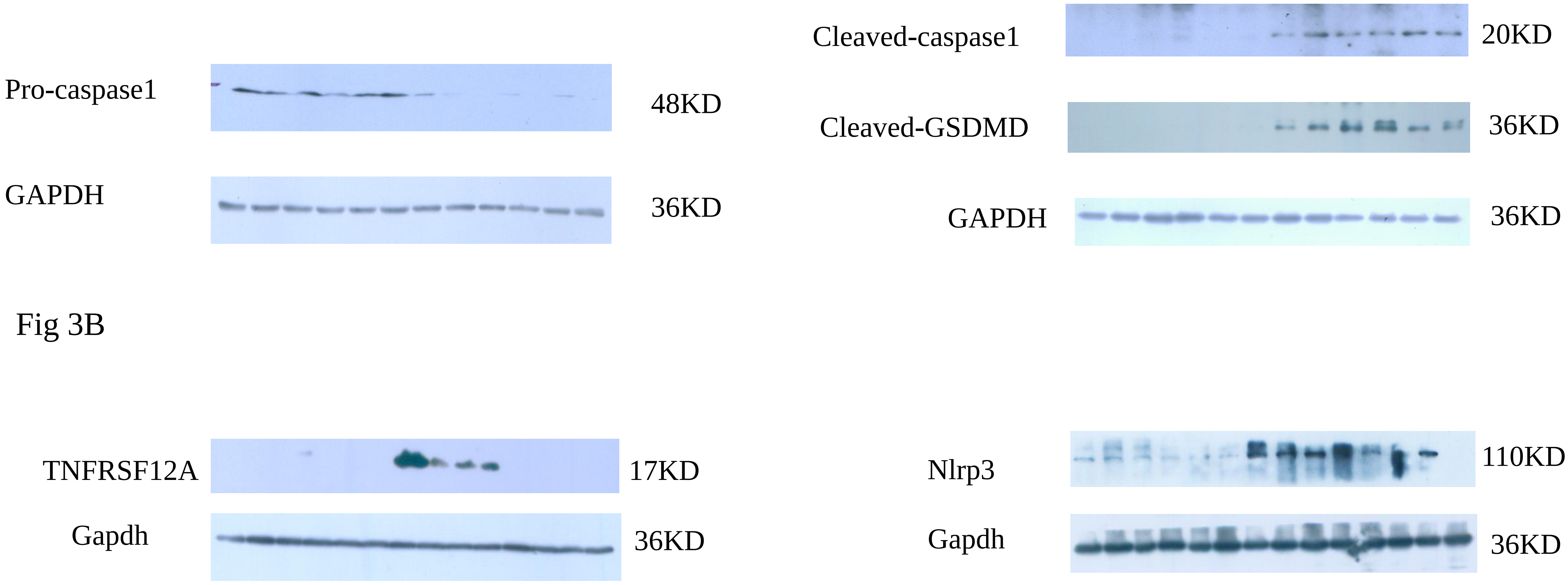
Pro-caspase1
48KD
GAPDH
36KD
Cleaved-caspase1
20KD
Cleaved-GSDMD
36KD
GAPDH
36KD
TNFRSF12A
17KD
Gapdh
36KD
Nlrp3
110KD
Gapdh
36KD

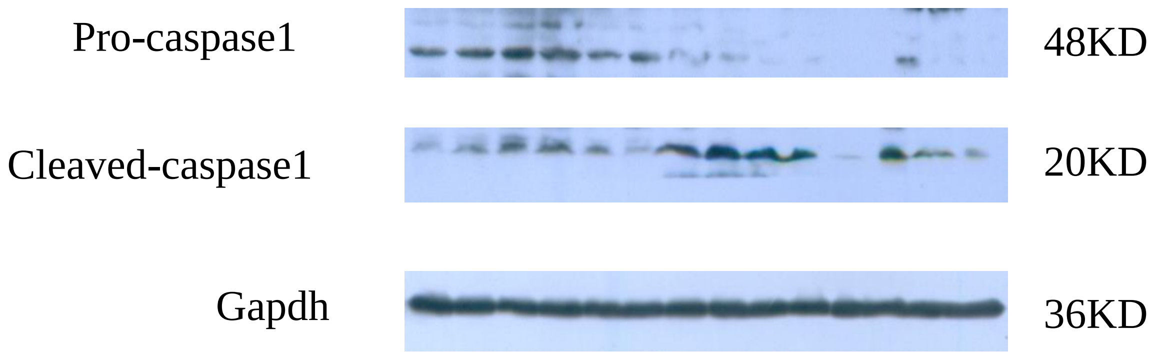
Pro-caspase1
48KD
Cleaved-caspase1
20KD
Gapdh
36KD

Fig 4B

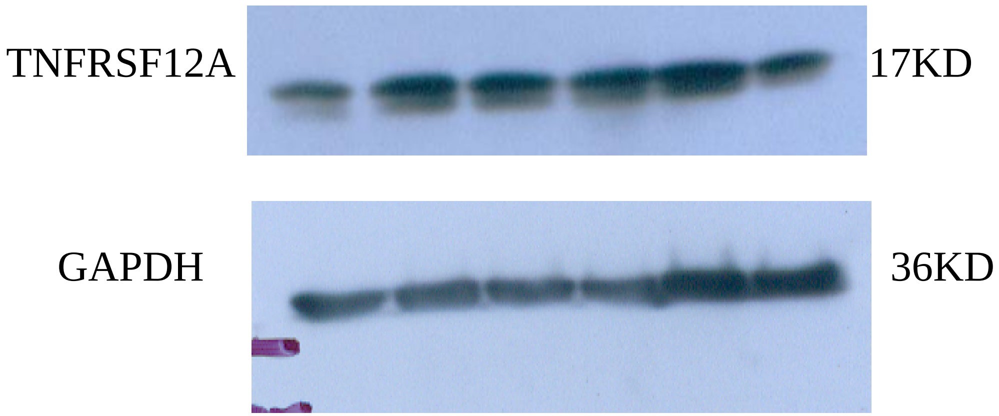
TNFRSF12A
17KD
GAPDH
36KD

Fig 4D

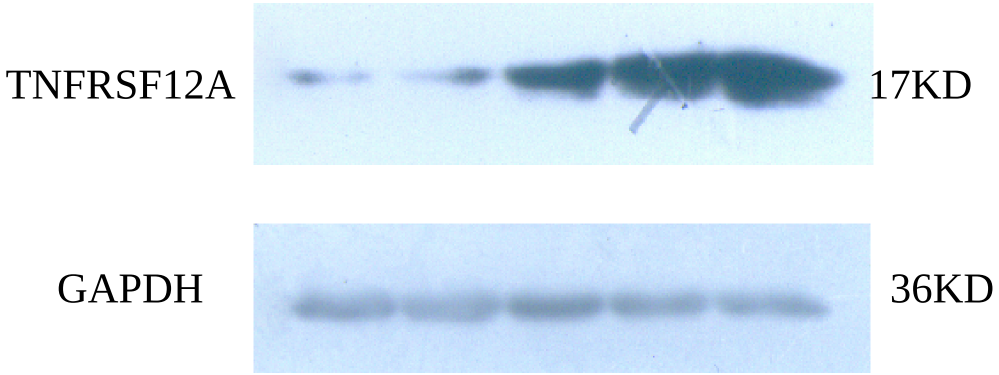
TNFRSF12A
17KD
GAPDH
36KD

Fig 4E

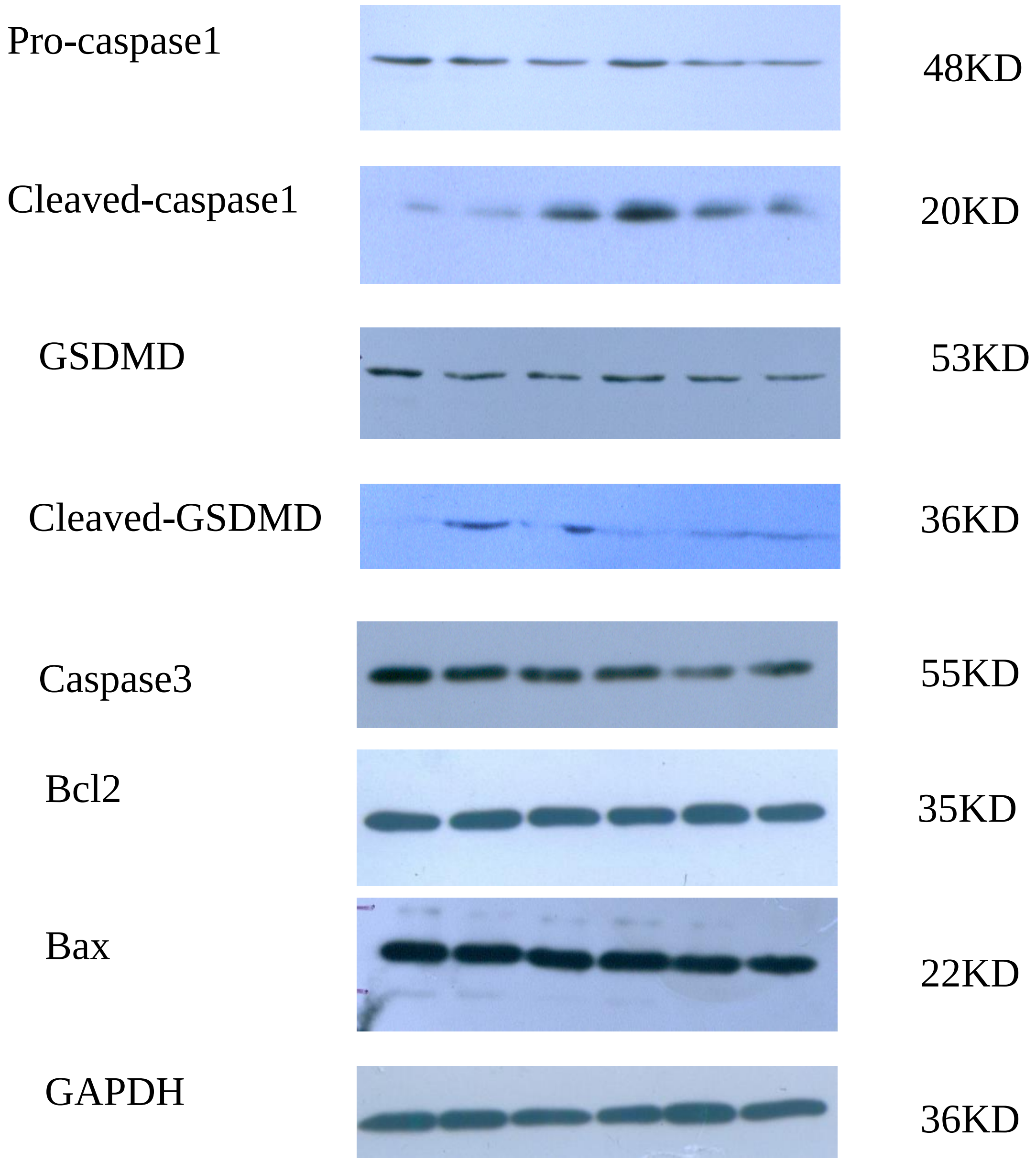
Pro-caspase1
48KD
Cleaved-caspase1
20KD
GSDMD
53KD
Cleaved-GSDMD
36KD
Caspase3
55KD
Bcl2
35KD
Bax
22KD
GAPDH
36KD

Fig 5A

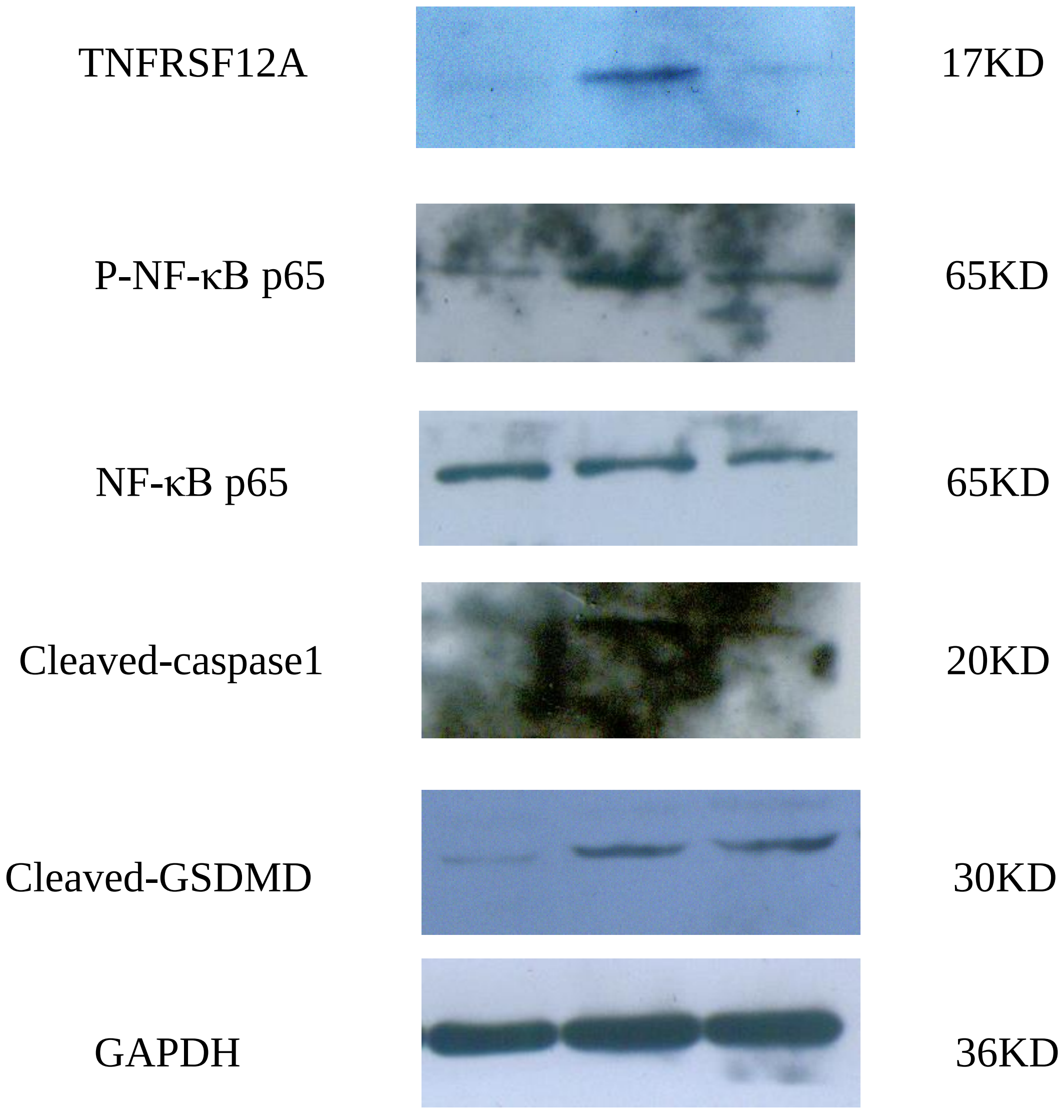
TNFRSF12A
17KD
P-NF-κB p65
65KD
NF-κB p65
65KD
Cleaved-caspase1
20KD
Cleaved-GSDMD
30KD
GAPDH
36KD

Fig 5B

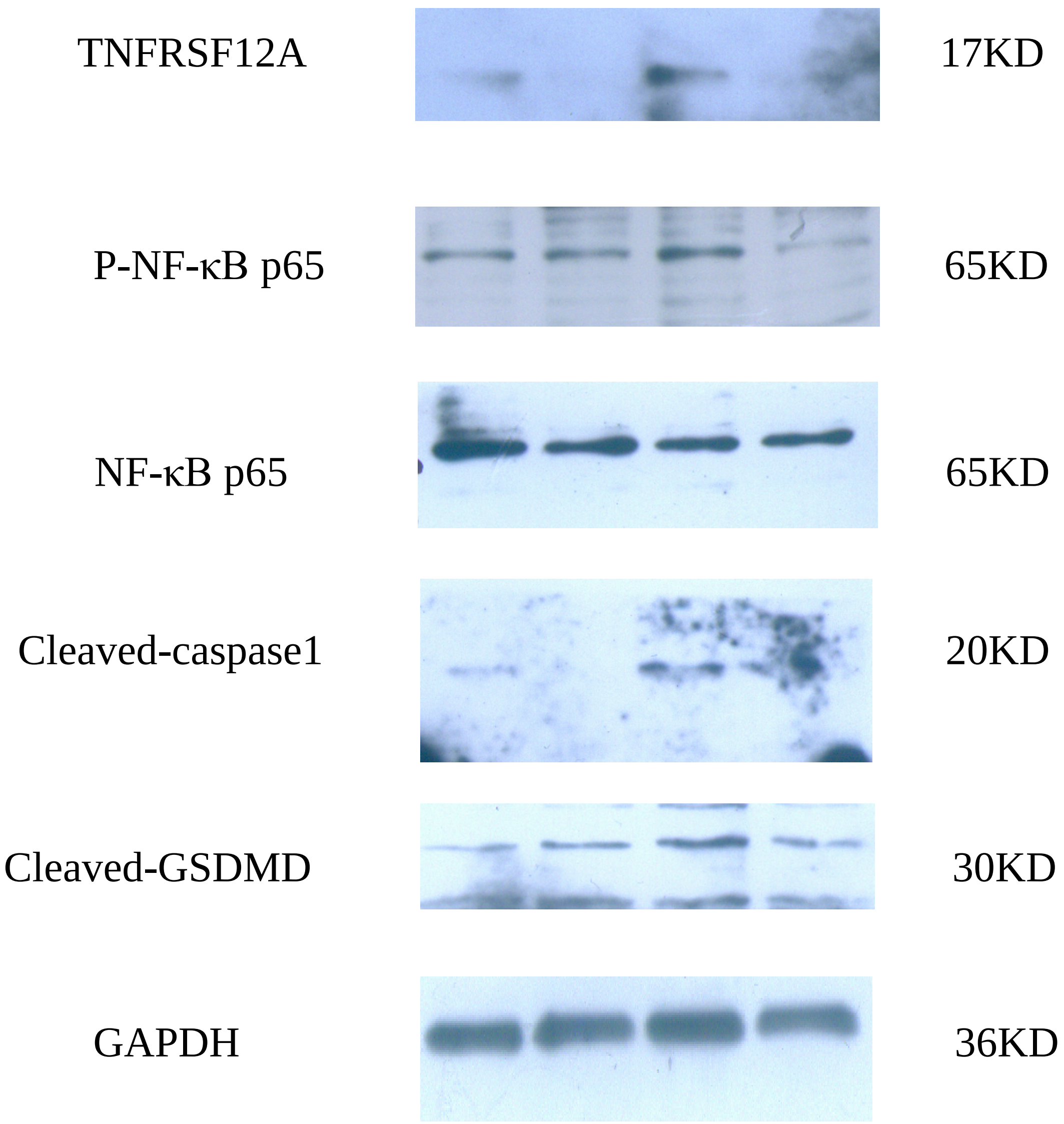
TNFRSF12A
17KD
P-NF-κB p65
65KD
NF-κB p65
65KD
Cleaved-caspase1
20KD
Cleaved-GSDMD
30KD
GAPDH
36KD

Fig 5C

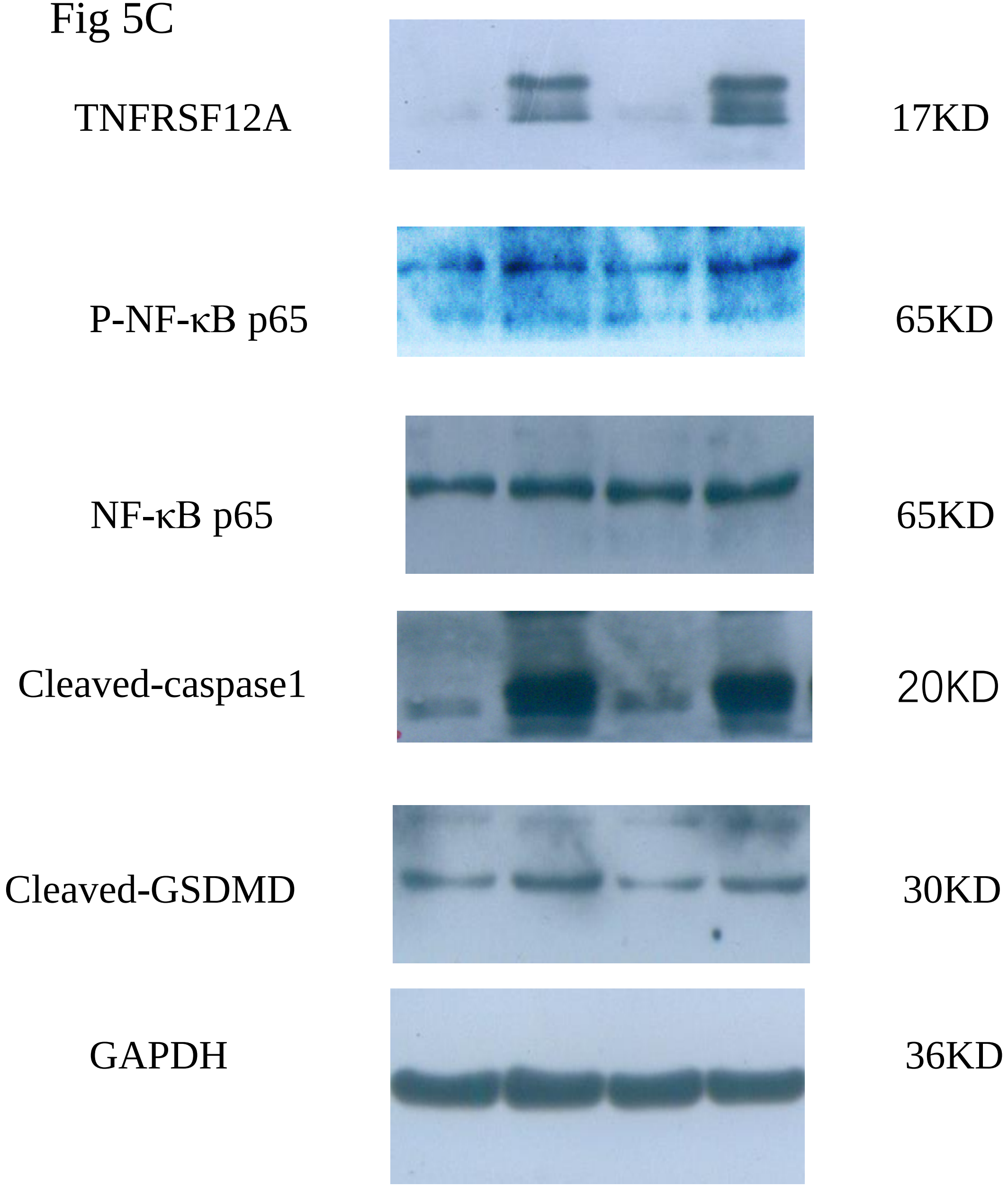
TNFRSF12A
17KD
P-NF-κB p65
65KD
NF-κB p65
65KD
Cleaved-caspase1
20KD
Cleaved-GSDMD
30KD
GAPDH
36KD

Fig 5D

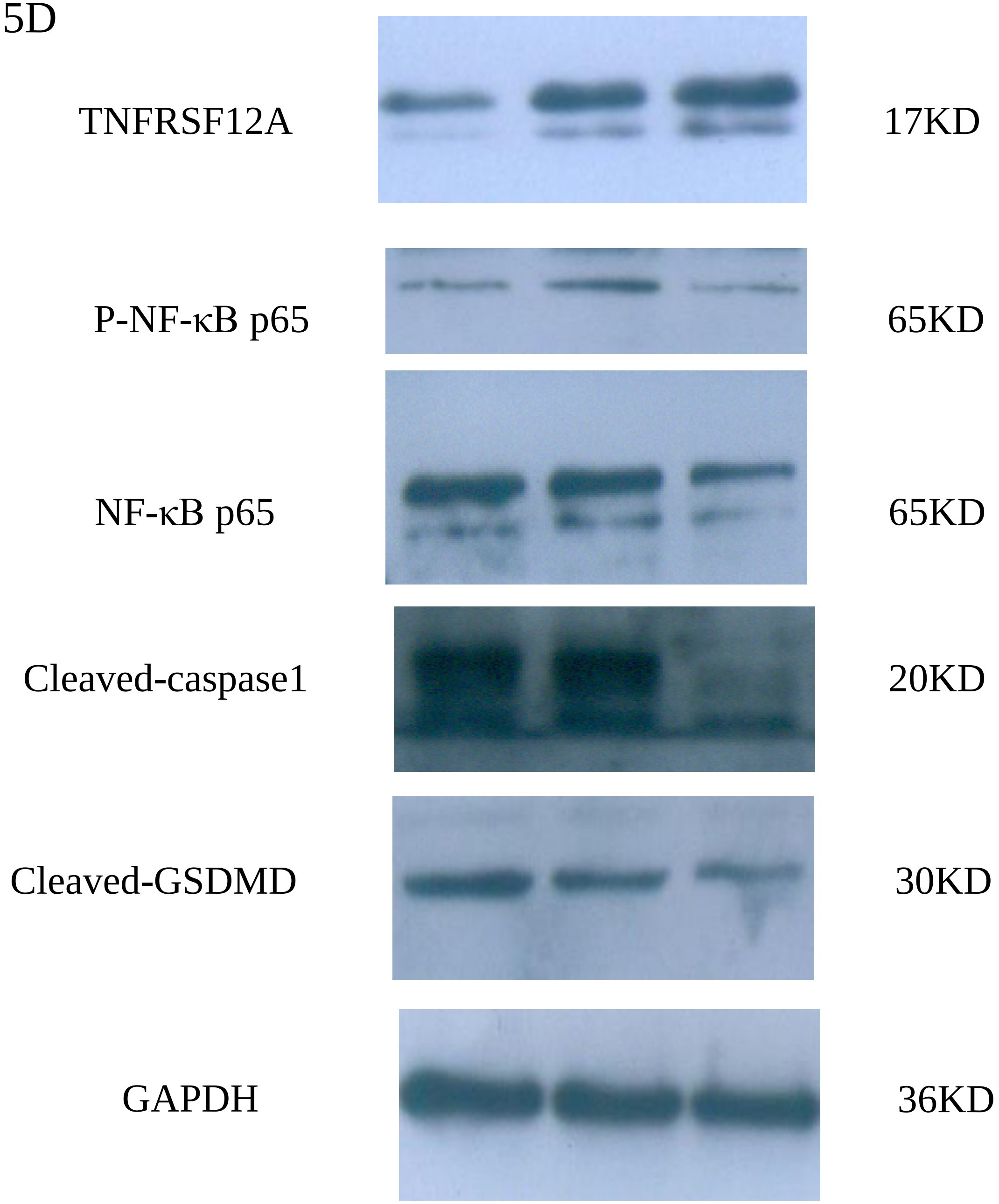
TNFRSF12A
17KD
P-NF-κB p65
65KD
NF-κB p65
65KD
Cleaved-caspase1
20KD
Cleaved-GSDMD
30KD
GAPDH
36KD

Fig 6H

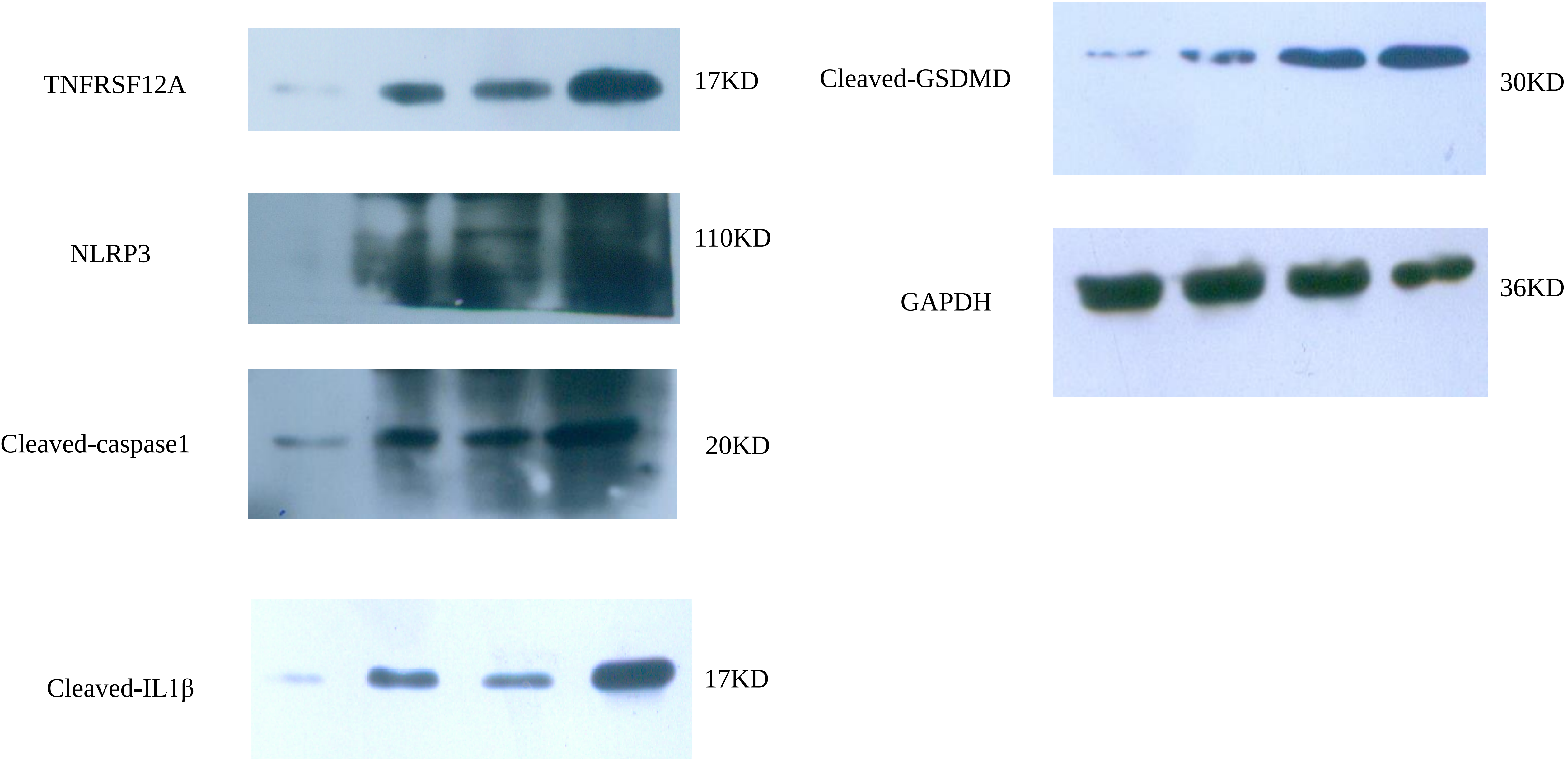
TNFRSF12A
17KD
NLRP3
110KD
Cleaved-caspase1
20KD
Cleaved-IL1β
17KD
Cleaved-GSDMD
30KD
GAPDH
36KD

Fig 7A

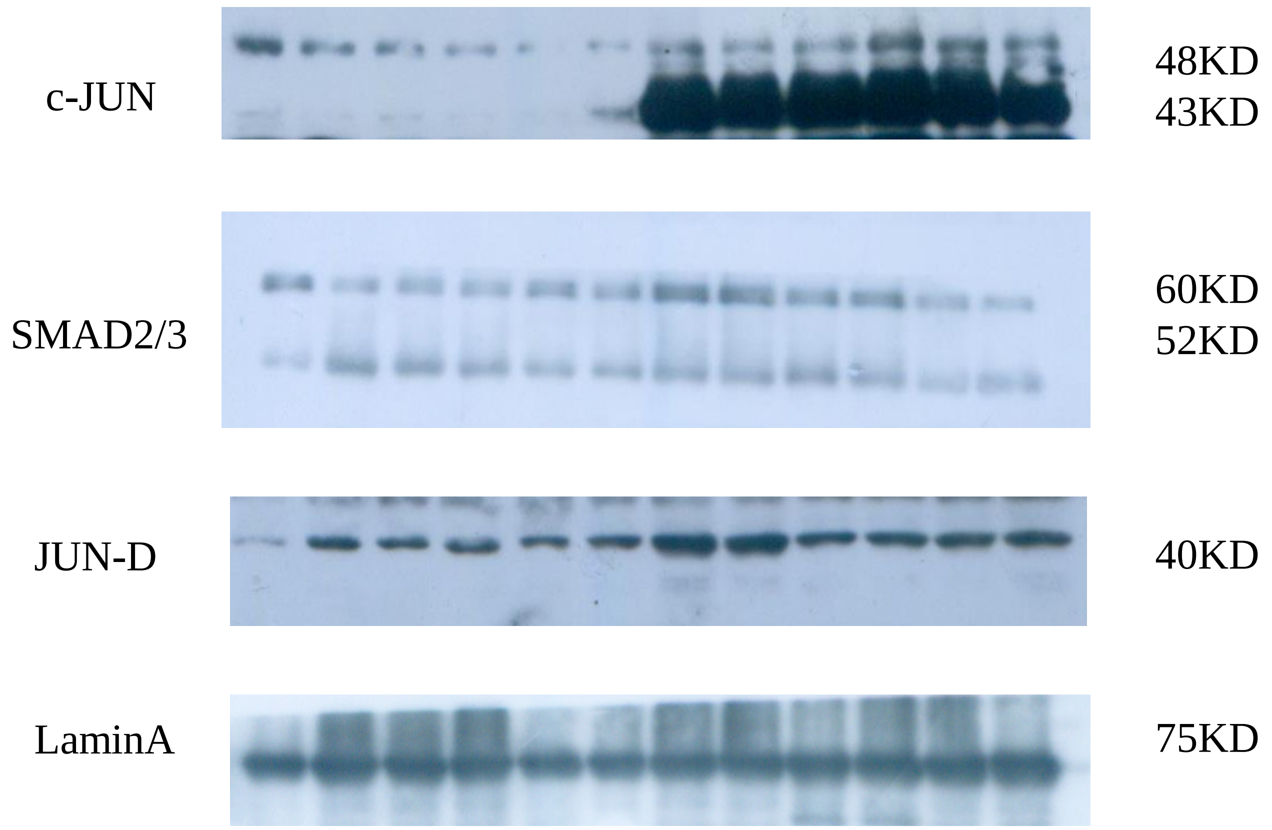

Fig 7B

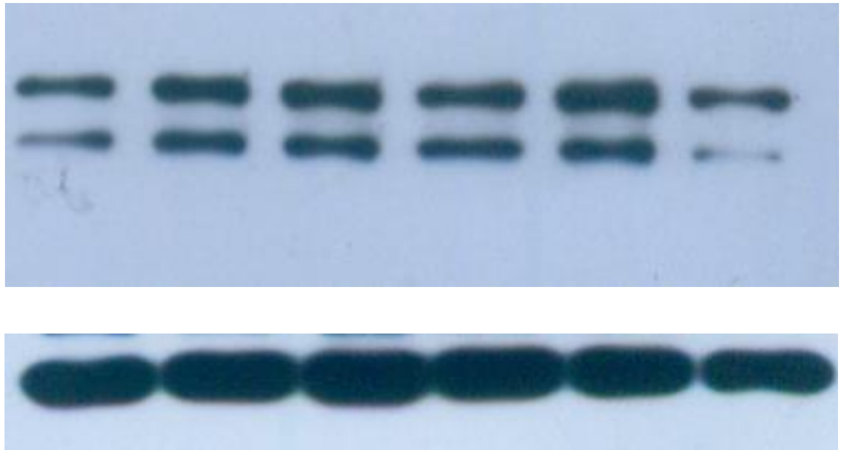

Fig 7C

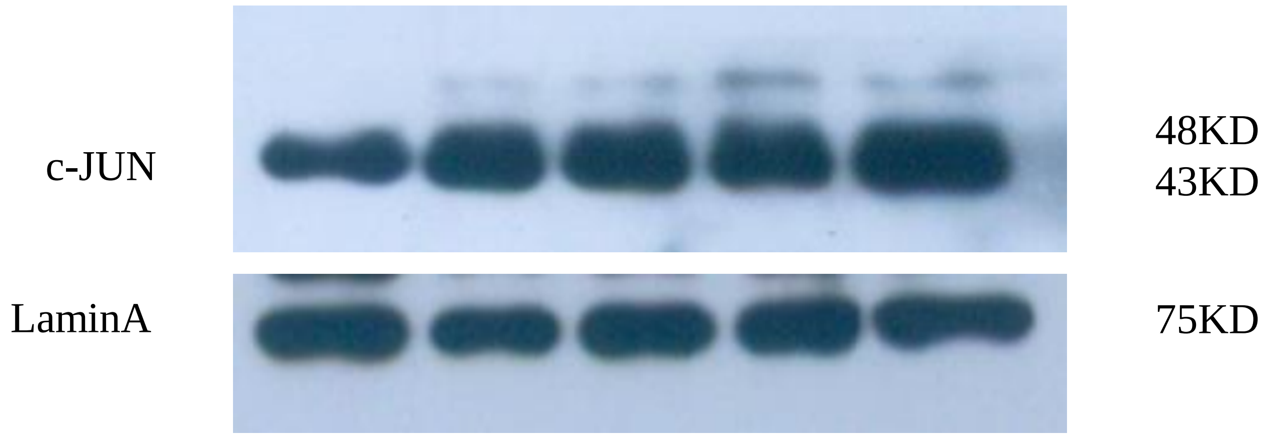

Supporting documents

Suppl. Figure 3C.

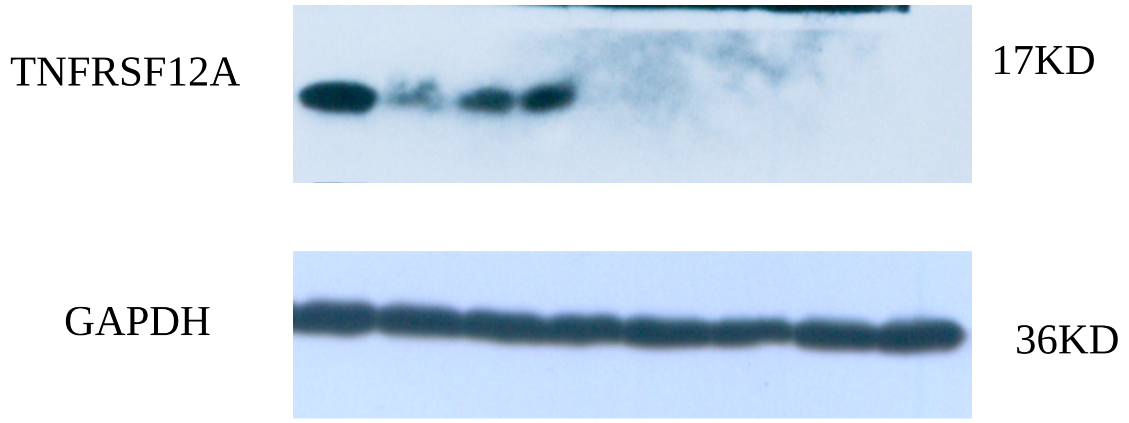

Supporting documents

Suppl. Figure 5A.

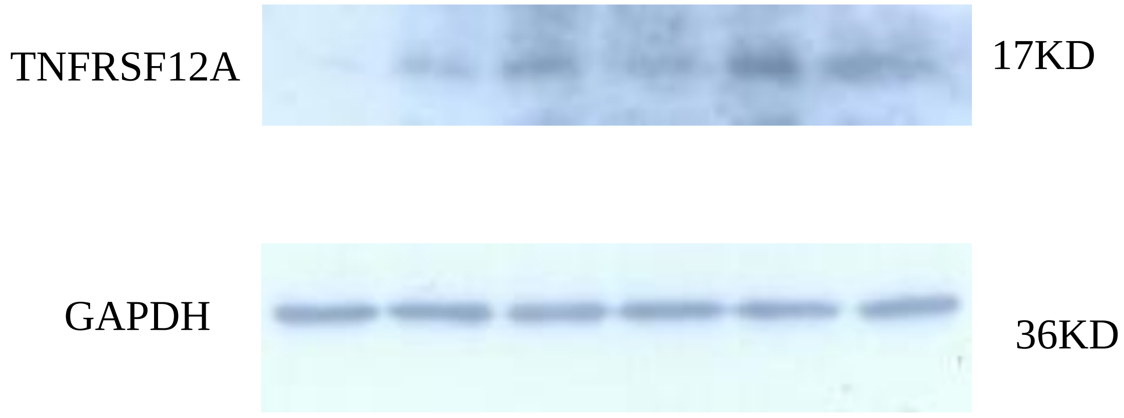

Suppl. Figure 5B.

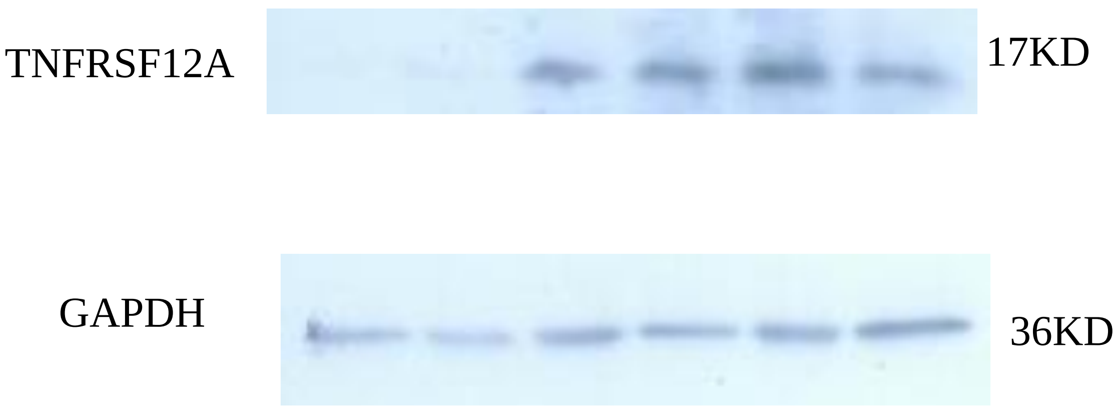

Supporting documents

Suppl. Figure 6.

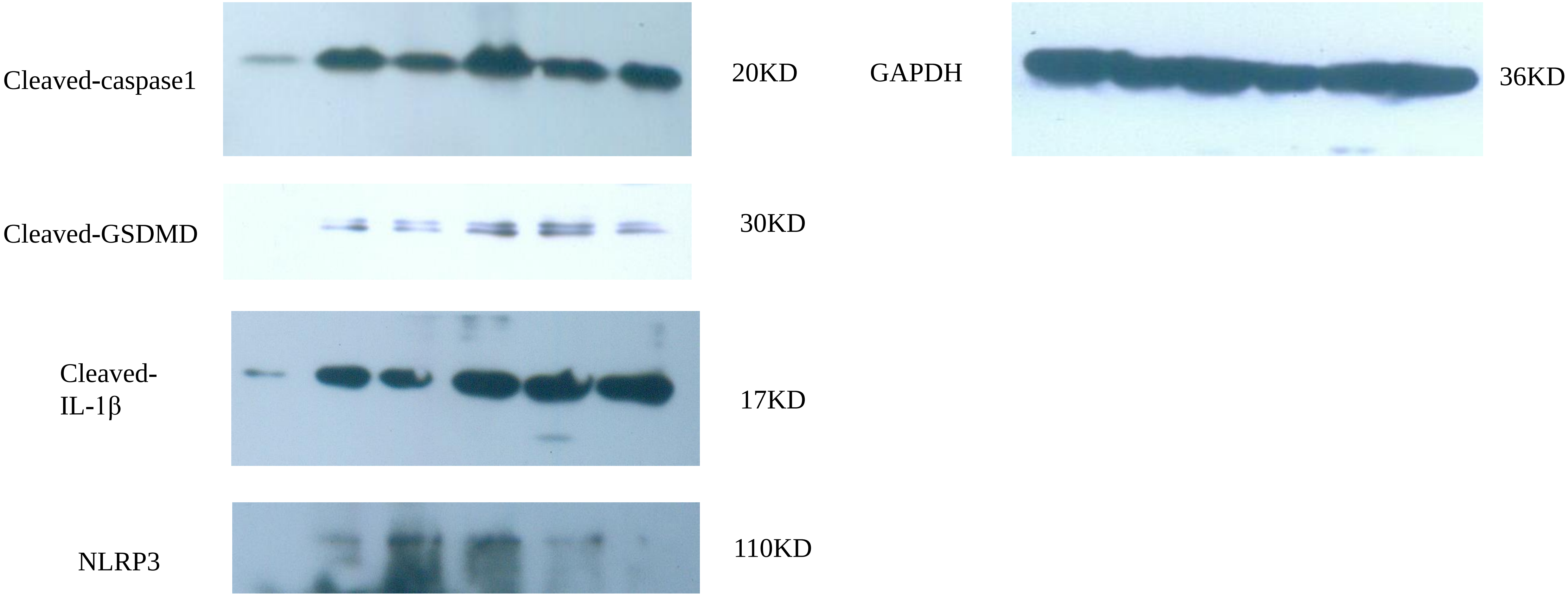

Supplement: Supplementary file 2 — Original Data File [file 41420_2023_1326_MOESM2_ESM.pdf]
